# CircAKT3 alleviates postoperative cognitive dysfunction by stabilizing the feedback cycle of miR-106a-5p/HDAC4/MEF2C axis in hippocampi of aged mice

**DOI:** 10.1007/s00018-024-05156-9

**Published:** 2024-03-13

**Authors:** Xuan Wang, Xiaole Tang, Pengfei Zhu, Dongyu Hua, Zheng Xie, Mingke Guo, Mengxin Que, Jing Yan, Xing Li, Qian Xia, Xiaoxiao Luo, Jiangjiang Bi, Yilin Zhao, Zhiqiang Zhou, Shiyong Li, Ailin Luo

**Affiliations:** 1 Department of Anesthesiology and Pain Medicine, Hubei Key Laboratory of Geriatric Anesthesia and Perioperative Brain Health, and Wuhan Clinical Research Center for Geriatric Anesthesia, Tongji Hospital, Tongji Medical College, Huazhong University of Science and Technology, 1095 Jiefang Avenue, Wuhan, 430030 Hubei China; 2 State Key Laboratory of Oncology in Southern China, Department of Anesthesiology, Sun Yat-Sen University Cancer Center, Guangzhou, 510060 Guangdong China; 3 Department of Oncology, Tongji Hospital, Tongji Medical College, Huazhong University of Science and Technology, Wuhan, 430030 China

**Keywords:** Perioperative neurocognitive disorders, circRNA, miRNA, Transcriptional regulation

## Abstract

Circular RNAs (circRNAs) have garnered significant attention in the field of neurodegenerative diseases including Alzheimer’s diseases due to their covalently closed loop structure. However, the involvement of circRNAs in postoperative cognitive dysfunction (POCD) is still largely unexplored. To identify the genes differentially expressed between non-POCD (NPOCD) and POCD mice, we conducted the whole transcriptome sequencing initially in this study. According to the expression profiles, we observed that circAKT3 was associated with hippocampal neuronal apoptosis in POCD mice. Moreover, we found that circAKT3 overexpression reduced apoptosis of hippocampal neurons and alleviated POCD. Subsequently, through bioinformatics analysis, our data showed that circAKT3 overexpression in vitro and in vivo elevated the abundance of miR-106a-5p significantly, resulting in a decrease of HDAC4 protein and an increase of MEF2C protein. Additionally, this effect of circAKT3 was blocked by miR-106a-5p inhibitor. Interestingly, MEF2C could activate the transcription of miR-106a-5p promoter and form a positive feedback loop. Therefore, our findings revealed more potential modulation ways between circRNA-miRNA and miRNA-mRNA, providing different directions and targets for preclinical studies of POCD.

**Supplementary Information:**

The online version contains supplementary material available at 10.1007/s00018-024-05156-9.

## Introduction

With the increasing global aging, the high incidence (9–54%) of postoperative cognitive dysfunction (POCD) poses a significant concern particularly in elderly patients [1]. POCD manifests as a decline in memory, attention disorder, and impaired language ability, which typically emerges following anesthesia and surgery [2]. POCD was linked to prolonged hospital stay, increased risk for mortality, and postoperative long-term cognitive impairment [3]. Previous studies mainly focus on exploring the mechanism of neuroinflammation, gut microbiota dysbiosis, disturbed energy metabolism of the brain, and altered neural circuits in POCD [4–7]. Currently accepted risk factors include advanced age and preoperative neurocognitive impairment [2]. Notably, the epigenetic mechanisms regulated by noncoding RNAs have been extensively investigated in relation to Alzheimer’s disease (AD) [8]. However, a large number of published research on POCD has only revealed the profiles of differentially expressed noncoding RNAs between POCD and control mice, warranting further exploration of the specific underlying mechanisms.

Different from other noncoding RNAs, circular RNAs (circRNAs) are not easily degraded due to their stable closed circular structure [9]. CircRNAs have been confirmed that act as sponges of microRNA (miRNA) or protein, interact with messenger RNA (mRNA), form R-loops to modulate transcription, and encode proteins [10]. However, the functions of circRNAs are still not fully revealed. MiRNAs typically interact with Argonaute (AGO) proteins to regulate the translation of mRNAs [11]. Messenger RNAs are translated into proteins, which participate in pathways to charge intracellular environment [12]. In view of the dynamic variability between pairwise regulation, the function of the circRNA-miRNA-mRNA network would be highly complex [10]. To acquire a comprehensive understanding of the involvement of circRNAs in both physiological and pathological states, whole-transcriptome sequencing is a preferable method for identifying changes in different RNAs at specific time points in vivo.

In our study, we initially compared the whole transcriptome profiles of the hippocampus between the POCD group and the normal group that consists of C57BL/6 male mice aged 18 months. Combined bioinformatics analysis with molecular biology experiments, we aimed to find the function of differentially expressed circRNA, miRNA, and mRNA. Based on the expression profiles and prediction of interactions between non-coding RNAs, specific circRNA (circAKT3) was selected and the downstream molecules miR-106a-5p/HDAC4/MEF2C were investigated. We explored the role of circAKT3/miRNA-106a-5p/HDAC4/MEF2C in neuronal apoptosis of POCD and validated the effect of MEF2C on transcriptional activation of miR-106a, which forms a feedback loop to amplify the impact of the pathway.

## Results

### Downregulated circAKT3 was accompanied by increased neuronal apoptosis in the hippocampi of mice with postoperative cognitive dysfunction

**Fig. 1:**
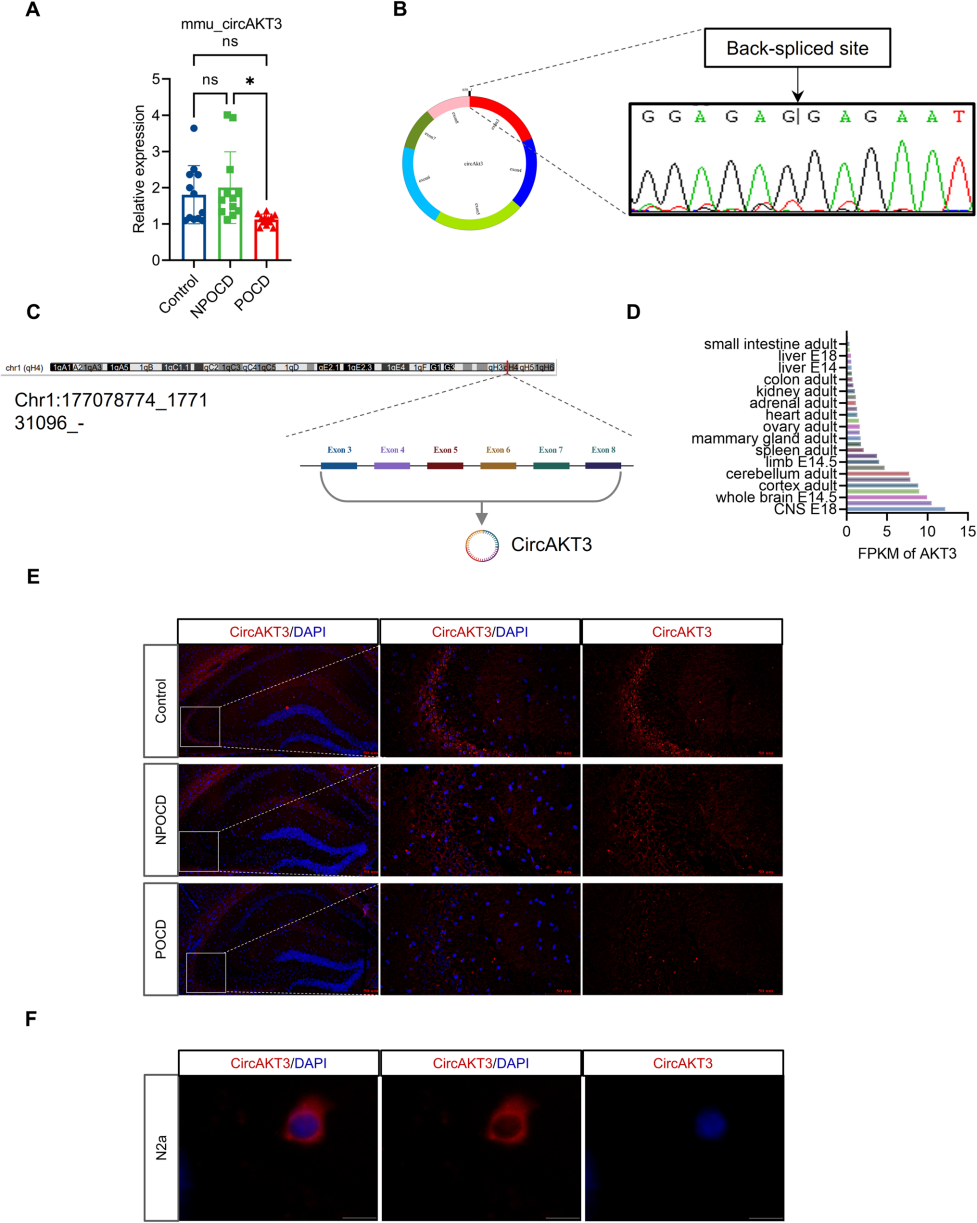
CircAKT3 is significantly decreased in the hippocampal CA3 region of mice with postoperative cognitive dysfunction (POCD). **A** CircAKT3 was reduced in the POCD mice compared to the non-POCD (NPOCD) and control group. **B** The circular structure of circAKT3 was drawn by circPrimer and validated by Sanger sequencing after the polymerase chain reaction of the back-spliced site. **C** CircAKT3 was generated from the third to the eighth exon of AKT3. The location of circAKT3 on the parental gene was produced by the National Center for Biotechnology Information (NCBI) and a schematic picture was drawn by BioRender. **D** The parental gene was predominantly expressed in the brain. The expression data was downloaded from the Gene database of NCBI and visualized by GraphPad Prism software. **E** Fluorescence in situ hybridization (FISH) revealed that circAKT3 was localized in the neuronal cytoplasm of the hippocampal CA3 region and circAKT3 was significantly decreased in the POCD group. (F) FISH of N2a cells also showed that circAKT3 was expressed mainly in the cytoplasm

A total of 52 aged mice underwent anesthesia with isoflurane and subsequently underwent intramedullary fixation surgery for open tibial fracture and behavioral tests (Fig. S1A). By employing the hierarchical cluster to analyze the results of the Morris water maze, the mice were categorized into two groups: postoperative cognitive dysfunction (POCD), non-POCD (NPOCD), and undetermined (Fig. S1B-K). To investigate the molecular mechanisms that underlie POCD, the hippocampi of selected mice from the POCD and NPOCD groups were subjected to whole transcriptome sequencing. Each sample comprised the hippocampi of two mice, with three samples tested for each group (Fig. S2A). The differentially expressed genes (DEGs) profiles were used to guide the selection of circRNAs, miRNAs, and mRNAs for subsequent quantitative real-time polymerase chain reaction (qRT-PCR) analysis, with or without RNase R digestion (Fig. S2B-G). Compared with the NPOCD group, circAKT3 was chosen due to its exponential decrease in the hippocampus of mice with POCD (Fig. 1A). To further validate the circular structure of circAKT3, sanger sequencing was performed using convergent primers across the back-spliced junction site (Fig. 1B).

**Fig. 2:**
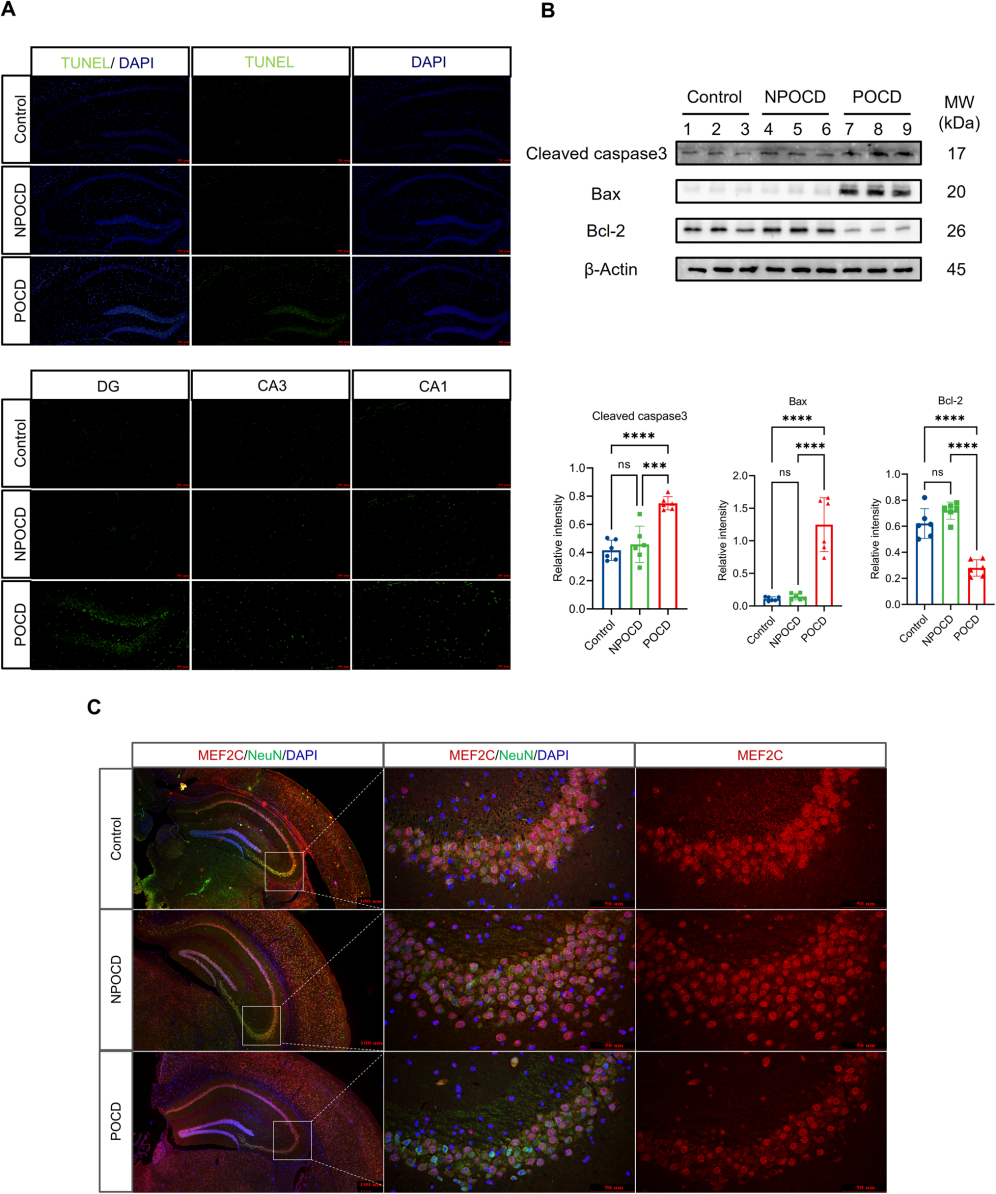
Increased Neuronal apoptosis and decreased MEF2C expression were observed in the hippocampus of POCD mice. **A** TUNEL staining showed an elevated apoptosis level in the hippocampus of the POCD group. **B** Western blot revealed the significantly decreased anti-apoptotic B-cell lymphoma 2 (BCL2) protein and increased pro-apoptotic cleaved caspase 3 protein as well as BCL2-associated X (Bax) protein in POCD mice, in comparison to NPOCD and control group. **C** Immunofluorescence showed a decrease in the fluorescence intensities in the hippocampal CA3 neurons of POCD mice. Scale bar, 50 μm or 100 μm as presented. The data was shown as mean ± SD. The P values were determined by one-way ANOVA with multiple comparison tests using GraphPad software 9.5 (**B**); *** *P* < 0.001, **** *P* < 0.0001, ns (not significant) means *P* > 0.05

CircAKT3 is a circular RNA derived from the third to the eighth exon of AKT serine/threonine kinase 3 (AKT3) (Fig. 1C). While the role of AKT3 in the hippocampus remains poorly understood, it is predominantly expressed in the brain [13], indicating that circAKT3 may have an essential function in the central nervous system [14] (Fig. 1D). To investigate the role of circAKT3 in POCD, we employed the fluorescence in situ hybridization (FISH) to demonstrate that circAKT3 is predominantly present in the cytoplasm of hippocampal neurons and Neuro-2a (N2a) cells (Fig. 1E, F). Furthermore, in order to identify potential competitive endogenous RNA (ceRNA) targets, a Kyoto Encyclopedia of Genes and Genomes (KEGG) pathway analysis was conducted. Notably, the MAPK signaling pathway was found to be enriched, and MEF2C mRNA was found to be involved in this pathway (Fig. S2H, I). Subsequently, in order to further investigate the involvement of circRNAs in POCD, we performed a Gene Ontology (GO) functional enrichment analysis of circRNA. Notably, the analysis also revealed that the differentially expressed circRNAs were enriched in the negative regulation of the MAPK signaling pathway and transcriptional regulation (Fig. S3A, B). We then detected the higher neuronal apoptosis of the hippocampus in the POCD group using TUNEL staining (Fig. 2A), which verified both enrichment analysis. Consistently, anti-apoptotic protein B-cell lymphoma 2 (BCL2) was reduced in the hippocampus of POCD mice (Fig. 2B). Pro-apoptotic proteins cleaved caspase 3 and BCL2-associated X protein (Bax) were increased (Fig. 2B). In addition, immunofluorescence of MEF2C indicated the decreased MEF2C protein in the hippocampal neurons of POCD mice (Fig. 2C). Next, we tested whether circAKT3 was involved in the modulation of hippocampal neuronal apoptosis.

### Overexpression of circAKT3 ameliorated neuronal apoptosis and postoperative cognitive dysfunction

**Fig. 3:**
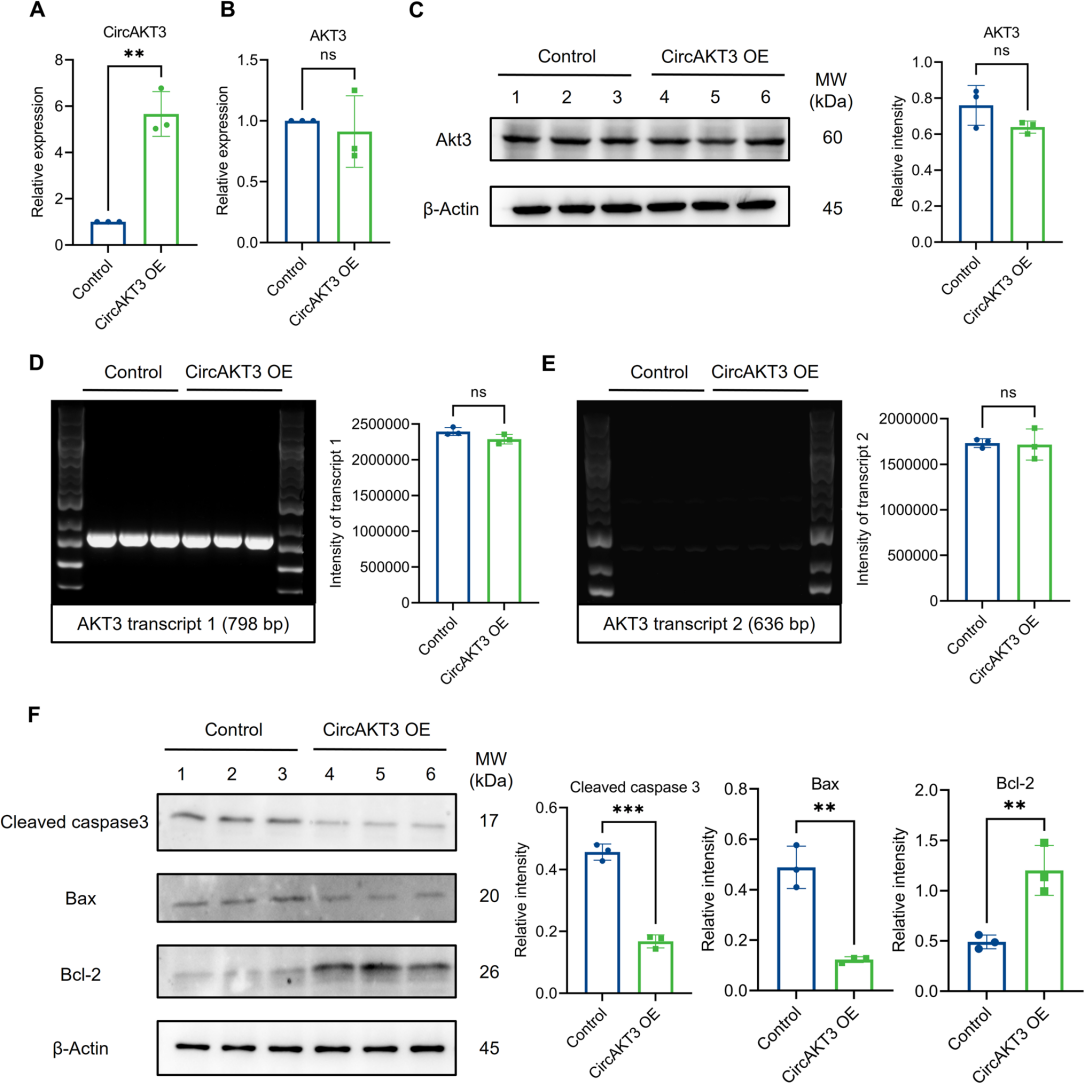
Overexpression of circAKT3 inhibited neuronal apoptosis in vitro. **A** Quantitative RT-PCR (qPCR) validated the overexpression of circAKT3 by lentivirus in N2a cells. **B** The interference on liner AKT3 messenger RNA of lentivirus was excluded by qPCR. **C** Western blot also validated that circAKT3 overexpression did not affect the expression of AKT3 protein. **D**, **E** Semi-quantitative RT-PCR indicated there was no significant difference in AKT3 transcript 1 (798 bp) and transcript 2 (636 bp) between the control and circAKT3 overexpression group. **F** The cleaved caspase 3 and Bax protein levels were decreased by circAKT3 overexpression (circAKT3 OE), while anti-apoptotic Bcl2 protein was increased. The *P* values were determined by two-tailed unpaired Student’s t-test; ** *P* < 0.01, *** *P* < 0.001, ns means *P* > 0.05

CircAKT3 exhibited a decrease in the neuronal cytoplasm of mice with POCD, particularly in the hippocampal CA3 region (Fig. 1E). In N2a cells, circAKT3 is also localized in the cytoplasm (Fig. 1F). In order to elucidate the impact of circAKT3 on neuronal apoptosis, we initially used lentivirus transfection to overexpress circAKT3 (CircAKT3-OE) in N2a cells. Western blot and qPCR were used to validate the transfection efficiency and exclude the interference of linear RNA (Fig. 3A–C). In detail, the host gene AKT3 has two isoforms formed by alternative splicing [15]. We also detected the level of AKT3 transcript 1 (also named protein kinase B γ) and transcript 2 (protein kinase B γ1) in the hippocampus of aged mice with RT PCR. Agarose gel electrophoresis revealed that circAKT3 overexpression did not affect the expression of AKT3 transcript 1 and transcript 2 (Fig. 3D, E). However, the further detection of isoforms of AKT3 protein was limited owing to the lack of commercial antibodies. Next, the expression of the anti-apoptotic BCL2 protein was significantly increased, while the pro-apoptotic proteins cleaved caspase 3 and Bax were downregulated upon circAKT3 overexpression (Fig. 3F). Consistently, the cell viability was notably higher at 24 h, 36 h, and 48 h in circAKT3-OE cells after Lipopolysaccharide (LPS) treatment (100 ng/ml, 12 h) compared to control cells (Fig. S3C).

**Fig. 4:**
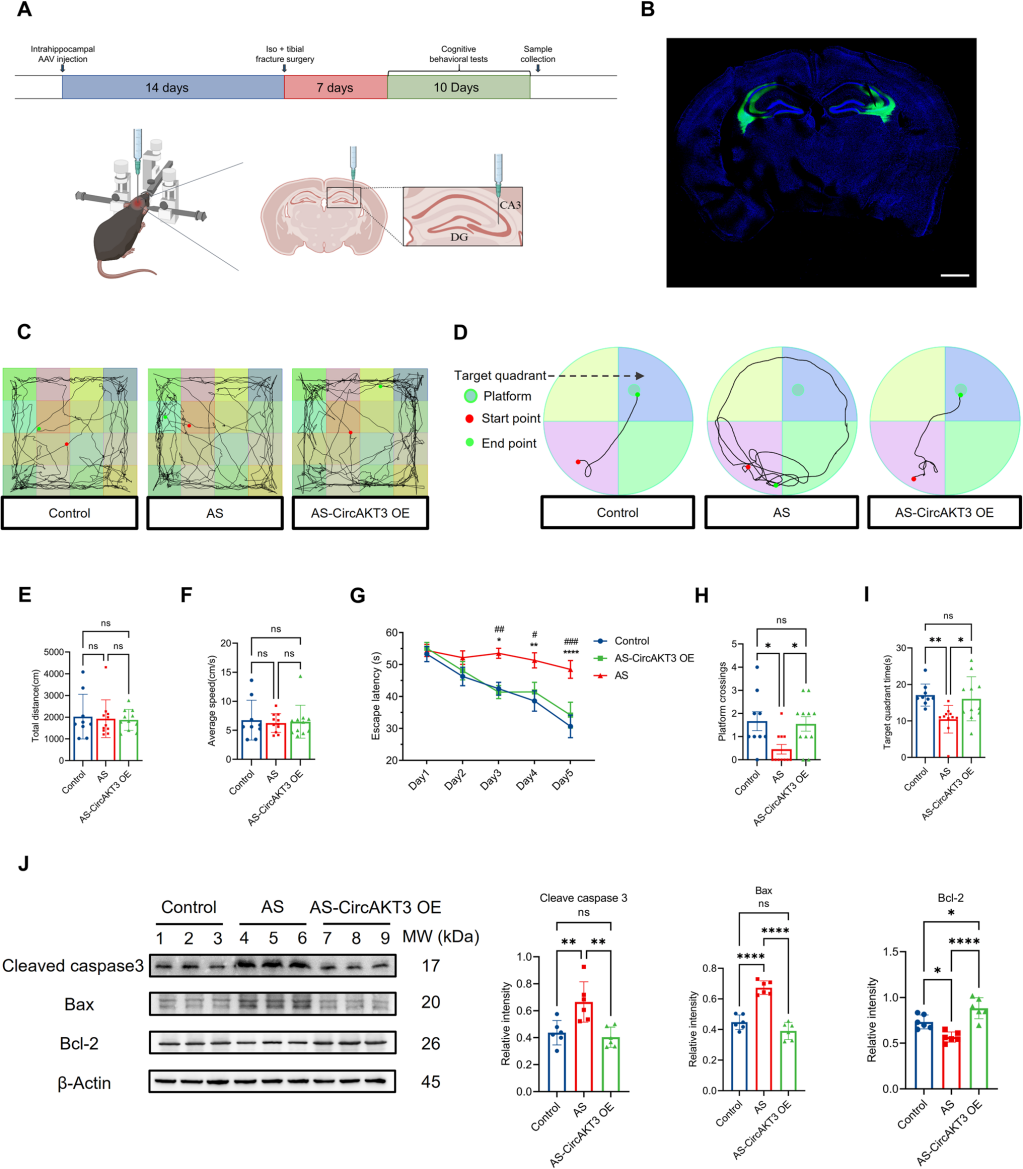
Overexpression of circAKT3 improved postoperative cognitive function and reduced the level of neuronal apoptosis in the hippocampal CA3 region after anesthesia and surgery. **A** The timeline and schematic picture of the experiment were drawn by BioRender. Following bilateral stereotactic injections in CA3 regions of elderly male mice, inhalatory anesthesia with isoflurane (iso) and tibial fracture surgery with intramedullary pinning (AS) were performed after 14 days. Then behavioral tests were conducted 7 days after surgery and anesthesia and lasted for 10 days, which was followed by sample collections of mice. **B** Immunofluorescence confirmed the overexpression of circAKT3 in bilateral hippocampal CA3 regions. **C** Open field tests for three groups including control, AS, and AS with circAKT3 overexpression (AS-CircAKT3 OE) groups were tracked and shown. **D** The tracks of the Morris water maze between three groups were shown. **E** There was no significant difference in the total distance traveled in the open field between the three groups. **F** The average speed of mice in the three groups also showed no difference in the open-field test. **G** The escape latency of the AS group was significantly higher than the control and AS-CircAKT3 OE group from day 3 to day 5 of the Morris water maze. Moreover, there was no difference between the control and AS-CircAKT3 OE groups. *n* = 9 (control group), *n* = 11 (AS and AS-CircAKT3 OE group). * *P* < 0.05 (control vs AS), ** *P* < 0.01 (control vs AS), # *P* < 0.05 (AS-CircAKT3 OE vs AS), ## *P* < 0.01 (AS-CircAKT3 OE vs AS). (H and I) AS group exhibited significantly decreased platform crossings and time spent in the target quadrant compared to the control and AS-CircAKT3 OE group. And AS-CircAKT3 group showed no difference with the control group. (J) Western blot revealed that there was a significantly decreased level of Bcl2 and a higher level of cleaved caspase 3 as well as Bax protein in the hippocampus of the AS group, which was reversed by circAKT3 overexpression. Scale bar (B) = 1000 μm. The *P* values were determined by one-way ANOVA (**E**, **F**, **H**–**J)** or two-way ANOVA (**G)** with multiple comparison tests; * *P* < 0.05, ** *P* < 0.01, **** *P < *0.0001, ns means *P* > 0.05 (**E**, **F**, **H**–**J**)

Additionally, to investigate whether circAKT3 could mitigate POCD and hippocampal neuronal apoptosis, we administered stereotaxic injections of recombinant adeno-associated viral vectors (rAAV) to overexpress circAKT3 (rAAV-hSyn-circAKT3-nEF1α-EGFP) or control vectors (rAAV-hSyn-nEF1α-EGFP) into the hippocampal CA3 region of aged mice two weeks prior to anesthesia and surgery (Fig. 4A). Immunofluorescence verified the overexpression and localization of circAKT3 in vivo (Fig. 4B). Performance of mice with circAKT3 overexpression in behavioral tests indicated a significant decrease in escape latency and an increased number of platform crossings during probe trials, with no impact on motor ability (Fig. 4C–I). Furthermore, overexpression of circAKT3 in vivo resulted in a reduction of hippocampal neuronal apoptosis detected by western blot (Fig. 4J), validating its ability to inhibit neuronal cell death and alleviate POCD. However, the downstream effectors of circAKT3 involved in improving cognitive performance are yet to be identified. Thus, we aim to explore the potential targets of circAKT3 in POCD mice in our subsequent studies.

### CircAKT3 bound to and elevated the level of miRNA-106a-5p in neuron

**Fig. 5:**
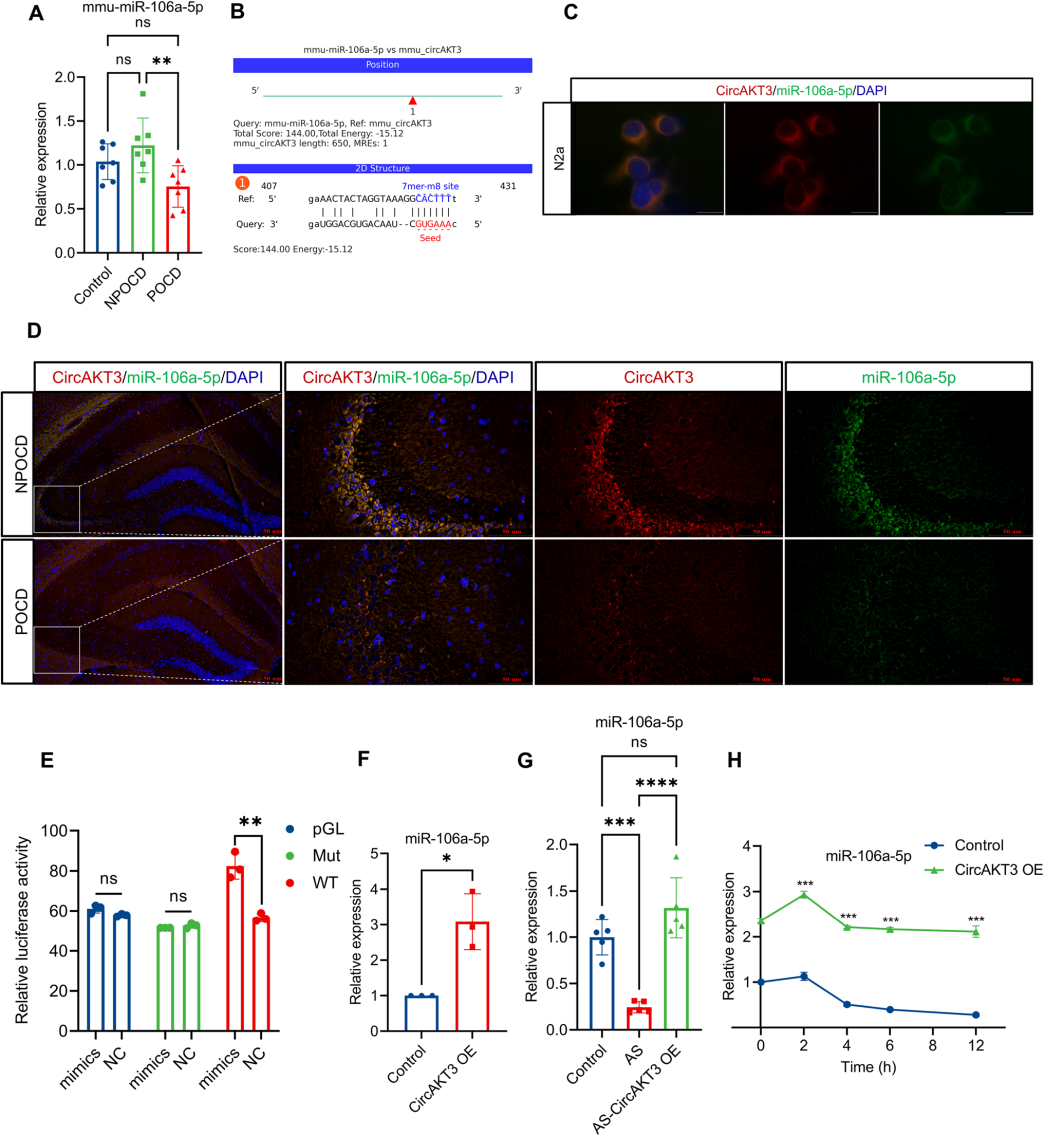
CircAKT3 could bind to miR-106a-5p and increase the level of miR-106a-5p by enhancing the stability of miR-106a-5p. **A** miR-106a-5p was remarkably decreased in the hippocampus of POCD mice compared to the NPOCD and control groups. **B** The potential binding site of circAKT3 and miR-106a-5p was predicted by Miranda and shown in the figure. **C** FISH revealed that miR-106a-5p was co-localized with circAKT3 in the cytoplasm of N2a cells. **D** FISH showed the co-localization of circAKT3 and miR-106a-5p in the neuronal cytoplasm of the hippocampus. Notably, both circAKT3 and miR-106a-5p were decreased in the neurons of the hippocampal CA3 region. **E** miR-106a-5p mimics significantly increased the relative luciferase activity of firefly luciferase plasmid containing the fragment of circAKT3 wild type (WT). However, mimics did not alter the activity of circAKT3 mutant type (Mut) luciferase plasmid or PGL3 plasmid. **F** CircAKT3 overexpression significantly elevated the level of miR-106a-5p in N2a cells. **G** Overexpression of circAKT3 in the neuron of the hippocampal CA3 region also increased the level of miR-106a-5p in neurons. **H** Actinomycin D treatment (10 μg/ml) in N2a cells inhibited the synthesis of new miR-106a-5p in the control group and circAKT3 OE group. CircAKT3 overexpression enhanced the stability of miR-106a-5p at 4, 6 and 12 h. The scale bar was 100 pixels (**C**) or 50 μm (**D**). The P values were determined by one-way ANOVA with multiple comparison tests (**A**, **G**), two-tailed unpaired Student’s t-test (E, F) or two-way ANOVA with multiple comparison tests (**H**); * *P* < 0.05, ** *P* < 0.01, *** *P* < 0.001, **** *P* < 0.0001, ns means *P* > 0.05

To identify the targets of circAKT3, we utilized miRanda to predict the potential interactions of circRNA/miRNA [16]. Furthermore, we compared the predicted interactions with the differentially expressed miRNA profiles between the NPOCD group and the POCD group. Our analysis revealed a significant downregulation of mmu-miRNA-106a-5p (miR-106a-5p) in the hippocampus of POCD mice (Fig. 5A). Moreover, Fig. 5B illustrates the possible complementary sites between circAKT3 and miR-106a-5p.

Next, FISH revealed that miR-106a-5p is mainly expressed in the cytoplasm of neurons in the hippocampal CA3 region and N2a cells (Fig. S3D, S4A). Additionally, miR-106a-5p was colocalized and in alignment with the expression of circAKT3 in N2a cells as well as hippocampal neurons between POCD and NPOCD mice (Fig. 5C, D). These findings suggested that circAKT3 may regulate neuronal transcription by interacting with miR-106a-5p. To validate this further, luciferase reporter plasmids containing wild-type (circAKT3-WT) or mutant-type (circAKT3-Mut) fragments of circAKT3 were constructed. Subsequent co-transfection of these plasmids along with miR-106a-5p mimics or negative control (NC) in 293T cells showed an increase of luciferase activity only in the circAKT3-WT group, indicating a specific interaction between miR-106a-5p and circAKT3 (Fig. 5E). Moreover, overexpression of circAKT3 in N2a cells led to a significant increase in miR-106a-5p levels (Fig. 5F). After anesthesia and surgery, the decrease of miR-106a-5p was also reversed by circAKT3 overexpression in the CA3 region of the hippocampus in vivo (Fig. 5G). To identify the detailed impact of circAKT3 on miR-106a-5p, we detected the stability of miR-106a-5p in N2a cells with the treatment of actinomycin D (10 μg/ml) for 0, 2, 4, 6 and 12 h (Fig. 5H). The half-life of miR-106a-5p was revealed as approximately 4 h and circAKT3 could enhance the stability of miR-106a-5p at 4, 6 and 12 h. These results robustly suggested that circAKT3 could bind to miR-106a-5p and enhance the expression of mature miR-106a-5p. However, it is worth noting that proteins are the primary mediators of cellular function. Therefore, in the next section, we investigated the downstream targets and the protein-related functions targeted by miR-106a-5p.

### miRNA-106a-5p promoted expression of MEF2C by inhibiting HDAC4 at posttranscriptional levels

**Fig. 6:**
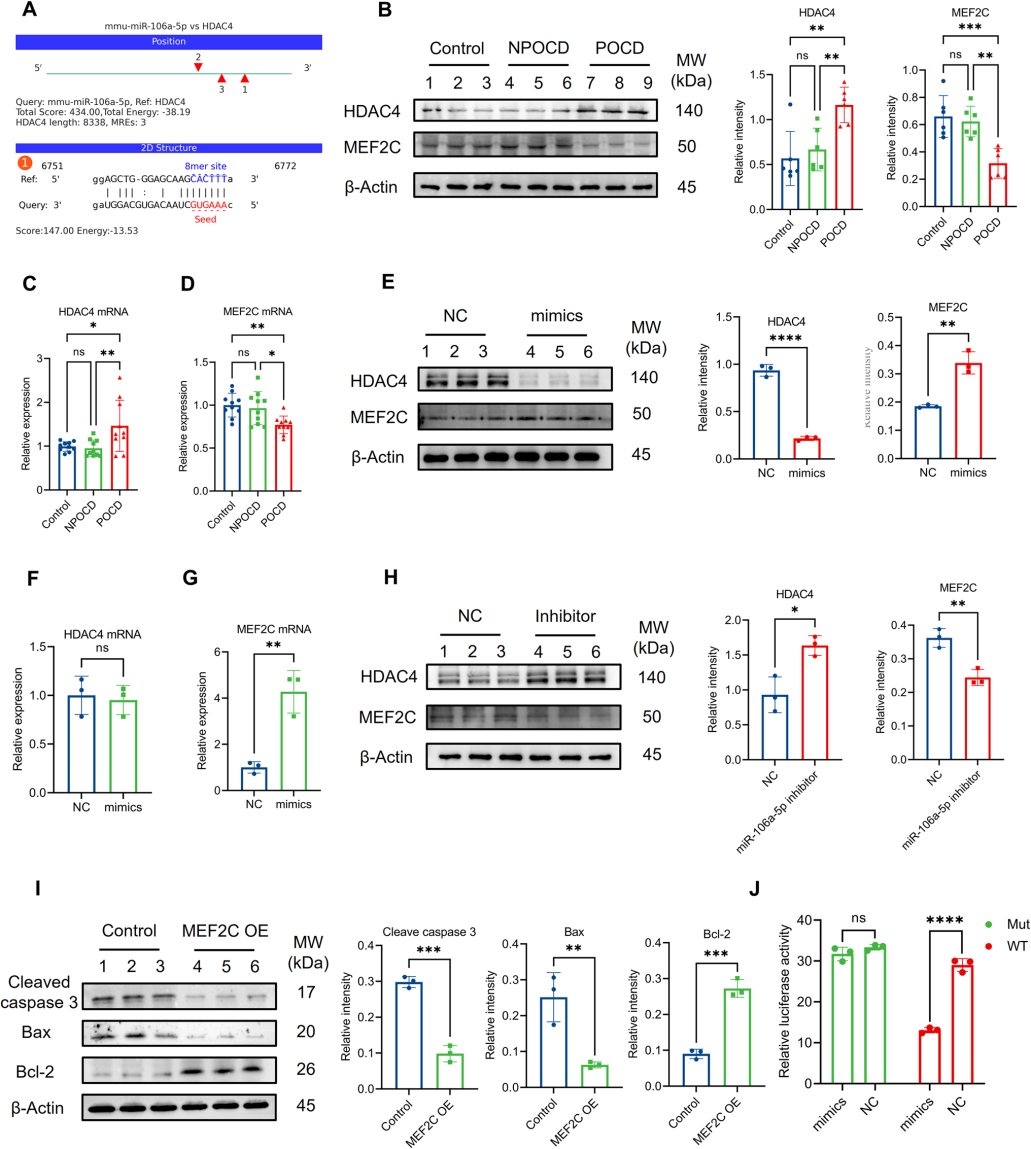
miR-106a-5p inhibited the expression of HDAC4 at the post-transcriptional level, then elevated the level of MEF2C protein. **A** The potential binding site predicted by Miranda between miR-106a-5p and HDAC4 was shown. **B**–**D** There was a decrease in both protein and mRNA of HDAC4 and an increase in MEF2C protein and mRNA in the POCD mice compared to NPOCD and control group. **E** miR-106a-5p mimics significantly reduced the level of HDAC4 protein and increased the level of MEF2C protein in N2a cells. **F** HDAC4 mRNA was not decreased after the transfection of miR-106a-5p mimics in comparison to the negative control (NC) group. **G** miR-106a-5p mimics significantly increased the level of MEF2C mRNA in N2a cells. **H** Conversely, the inhibitor of miR-106a-5p significantly increased the level of HDAC4 protein but reduced the level of MEF2C protein compared to the NC cells. **I** Western blot revealed that there was a significantly increased level of Bcl2 protein and a lower level of cleaved caspase 3 as well as Bax protein in N2a cells with MEF2C overexpression. **J** The relative luciferase activity of firefly luciferase plasmid containing HDAC4-WT segment was inhibited by miR-106a-5p mimics but not NC. Furthermore, there was no significant difference in luciferase plasmid containing HDAC4-Mut segment co-transfected with mimics or NC. The *P* values were determined by one-way ANOVA with multiple comparison test (**B**–**D**) or two-tailed unpaired Student’s *t* test (**E**–**I**); * *P* < 0.05, ** *P* < 0.01, **** *P* < 0.0001, ns means *P* > 0.05

Firstly, we performed a KEGG pathway enrichment analysis of genes targeted by differentially expressed miRNA utilizing R and applying the hypergeometric distribution. According to the fold change and KEGG classification, the pathway of environmental information processing (signal transduction) presented a significant enrichment (Fig. S4B, C). Additionally, the analysis of biological processes using GO enrichment revealed the regulation of transcription with DNA templates, indicating the potential involvement of epigenetic regulation including transcription factors (Fig. S4D). Based on our sequencing files, histone deacetylase 4 (HDAC4) was identified as the candidate target gene of miR-106a-5p. The binding sites predicted by Miranda are presented in Fig. 6A. Notably, HDAC4 is known as a class II histone deacetylase that has been reported to repress the activity of MEF2C through the formation of HDAC4/MEF2C complexes [17]. String also revealed the potential association of HDAC4 and MEF2C (Fig. S4E). We further examined the expression levels of HDAC4 and MEF2C in the hippocampi of POCD mice, and as shown in Fig. 6B–D, the protein and mRNA levels of HDAC4 were remarkably elevated, while MEF2C were respectively decreased.

To explore the regulatory effect of miR-106a-5p on HDAC4 and MEF2C, we transfected mimics or NC into N2a cells for a duration of 6 h. Subsequently, we measured the levels of HDAC4 and MEF2C mRNA and protein 48 h after transfection. Our results showed that miR-106a-5p mimics greatly decreased the level of HDAC4 protein and increased the level of MEF2C protein (Fig. 6E). However, the mRNA expression of HDAC4 exhibited a non-significant trend due to incomplete complementarity of the base sequence (Fig. 6F). MEF2C mRNA exhibited a significant increase after transfection of mimics (Fig. 6G). Furthermore, we repeated the transfection experiment using primary hippocampal neurons and observed consistent results in terms of HDAC4 and MEF2C protein as well as mRNA levels, as determined by qPCR and immunofluorescence (Fig. S5A-E).

In addition, the N2a cells were transfected with the inhibitor of miR-106a-5p. As a result, a significant increase of HDAC4 protein and a reduction of MEF2C protein was observed (Fig. 6H). Consistently, anti-apoptotic protein BCL2 was increased and pro-apoptotic protein BAX and Cleaved caspase3 were reduced in N2a cells after transfection with MEF2C plasmids (Fig. 6I). To further confirm the binding sites, Firefly luciferase reporter plasmids were constructed with the wild-type or mutant-type HDAC4 fragments. These Firefly luciferase reporters were then co-transfected with miR-106a-5p mimics or NC, together with Renilla luciferase. We found that miR-106a-5p mimics decreased the luciferase activity of the HDAC4-WT luciferase reporter, but failed to reduce the activity of the HDAC4-Mut luciferase reporter (Fig. 6J). Therefore, miR-106a-5p reduced HDAC4 protein levels at the posttranscriptional level and promoted the elevation of MEF2C protein. This finding raised the question of whether circAKT3 regulates the expression of HDAC4 and MEF2C by acting on miR-106a-5p.

### CircAKT3 inhibited HDAC4/MEF2C by increasing miRNA-106a-5p level

**Fig. 7:**
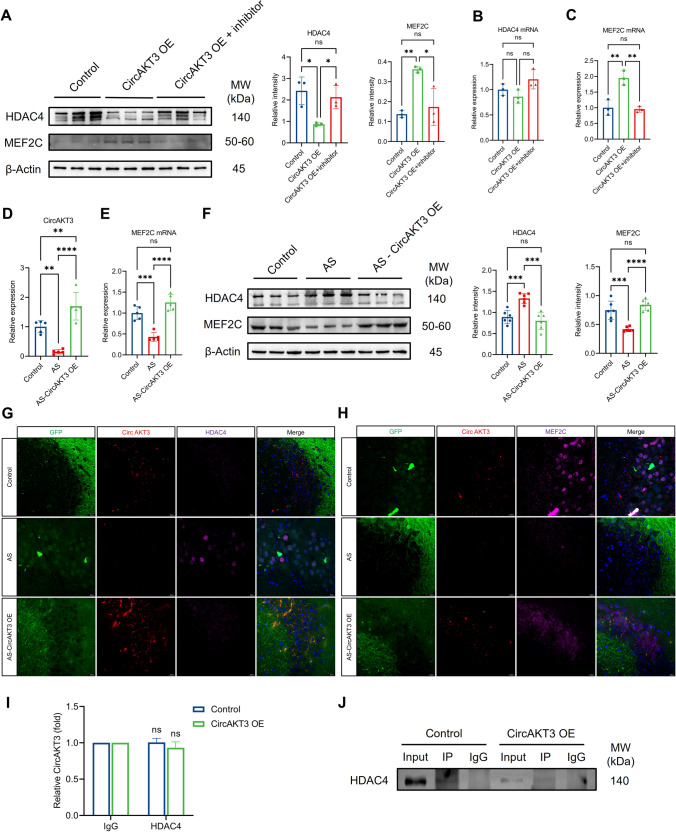
CircAKT3 inhibited the expression of HDAC4 protein and elevated the level of MEF2C protein by increasing the level of miR-106a-5p. **A** The overexpression of circAKT3 markedly reduced the level of HDAC4 protein and increased the level of MEF2C protein in N2a cells. This effect was blocked by the inhibitor of miR-106a-5p. **B** There was no significant difference in HDAC4 mRNA between the three groups. **C** MEF2C mRNA was notably increased after the overexpression of circAKT3, which was further reversed by miR-106a-5p inhibitor. **D** CircAKT3 was significantly decreased in the hippocampus of mice with anesthesia and surgery (AS). Additionally, there was a significant increase of circAKT3 in the mice that underwent circAKT3 overexpression in hippocampal CA3 neurons following anesthesia and surgery (AS-CircAKT3 OE). **E** The level of MEF2C mRNA was reduced in the AS group, which was reversed in the AS-CircAKT3 OE group. **F** The significant increase of HDAC4 protein and reduction of MEF2C protein in the hippocampus of AS mice were reversed by circAKT3 overexpression in vivo. (**G** and **H**) Immunofluorescence showed that circAKT3 overexpression in vivo with AS reduced the nuclear localization of HDAC4 protein but increased the expression of MEF2C protein in both the nucleus and cytoplasm compared to the AS group. **I**, **J** RNA immunoprecipitation showed there was no direct binding between circAKT3 and HDAC4 protein. Compared to the anti-IgG group, the anti-HDAC4 group showed no higher level of circAKT3 (**I**). ns means *P* > 0.05 (anti-HDAC4 group vs anti-IgG group). Western blot validated the specificity and binding affinity of primary antibodies with magnetic beads for RNA immunoprecipitation (**J**). The scale bar was 20 μm (**G**, **H**). The *P* values were determined by one-way ANOVA with multiple comparison tests; * *P* < 0.05, ** *P* < 0.01, **** *P* < 0.0001, ns means *P* > 0.05

In the presence of associations between circAKT3/miR-106a-5p and miR-106a-5p/HDAC4/MEF2C, the effect of circAKT3 on HDAC4/MEF2C need to be further explored. First, circAKT3 was overexpressed using lentivirus in N2a cells, leading to a significant decrease in HDAC4 protein levels and an increase in MEF2C protein levels (Fig. 7A). However, this effect was abolished when the miR-106a-5p inhibitor was applied, suggesting that miR-106a-5p at least partially mediates the effect of circAKT3 on HDAC4/MEF2C (Fig. 7A). There was a significant reduction in MEF2C mRNA but only an increased trend of HDAC4 mRNA after circAKT3 overexpression, which was further blocked by miR-106a-5p inhibitor (Fig. 7B, C).

Furthermore, overexpression of circAKT3 in hippocampal neurons of the CA3 region in vivo not only increased the levels of miR-106a-5p (Fig. 5G) but also raised MEF2C mRNA as well as protein levels by decreasing the expression of HDAC4 protein (Fig. 7D–F). What’s more, circAKT3 overexpression facilitated the nuclear localization of MEF2C protein and reduced the expression of HDAC4 both in the cytoplasm and nucleus (Fig. 7G, H). We postulate that the decreased HDAC4 proteins release more MEF2C by the formation of fewer HDAC4/MEF2C complexes, leading to the activation of neuronal transcription. As a transcription factor, MEF2C plays a significant role in regulating cognitive resilience and neuronal apoptosis [18–20]. In response to the reduced HDAC4 protein, increased nuclear MEF2C likely activates the transcription of genes involved in hippocampus-dependent learning and memory. We have also validated the anti-apoptotic effect of MEF2C in N2a cells (Fig. 6I). Additionally, we noticed that the modulation of miR-106a-5p/HDAC4 via circAKT3 differs from typical ceRNA networks. To further investigate potential explanations, the possibility of whether circAKT3 directly binds HDAC4 protein should be clarified first. Therefore we performed the RNA immunoprecipitation (RIP) assay and found there was no binding between circAKT3 and HDAC4 protein (Fig. 7I, J). And then we hypothesized that MEF2C could regulate the transcription of miR-106a-5p and form a positive feedback loop.

### MEF2C promoted the transcription of miRNA-106a-5p as a feedback loop

**Fig. 8:**
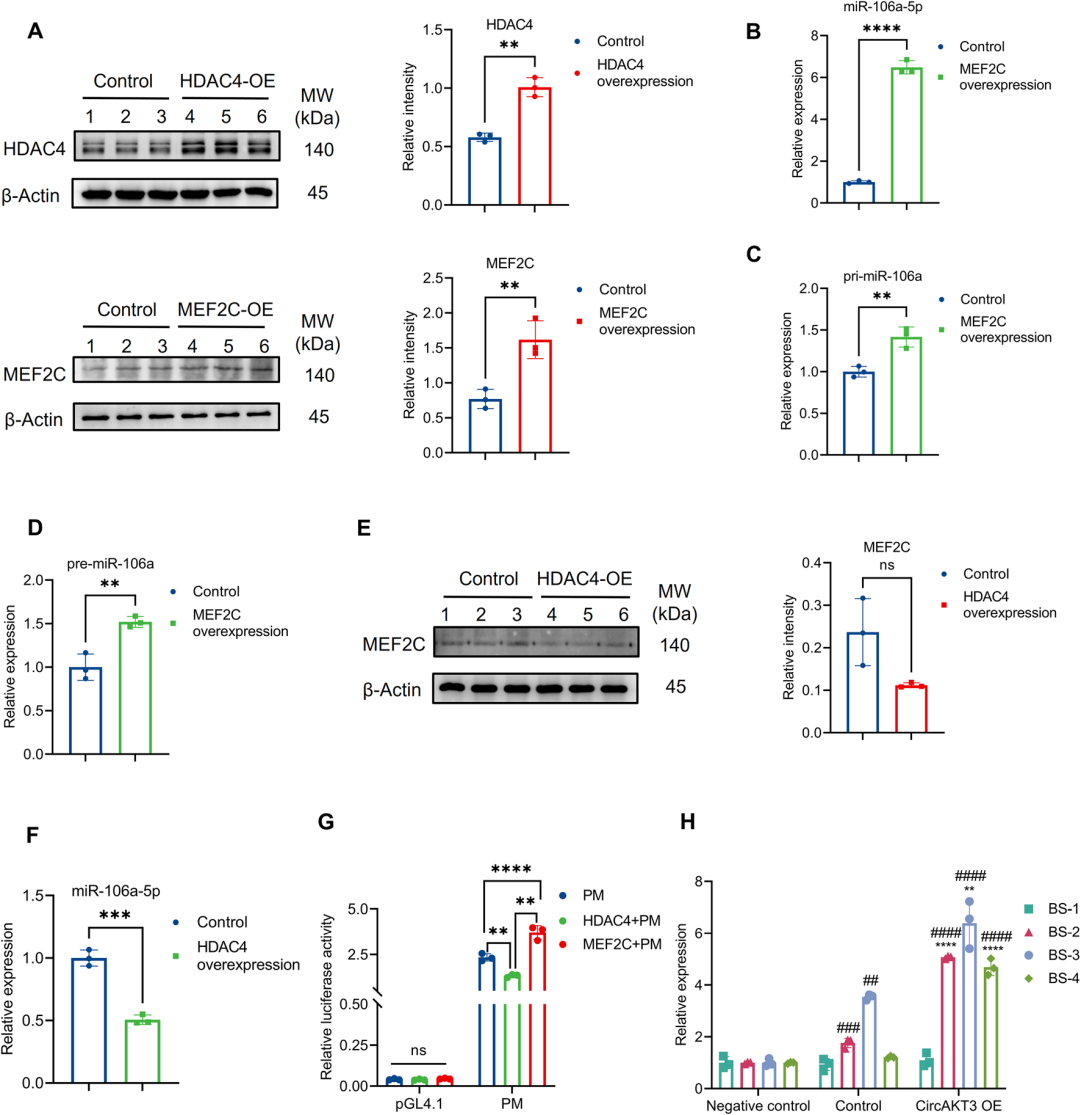
MEF2C promoted the transcription of miR-106a-5p and HDAC4 inhibited the transcription of miR-106a-5p by reducing the level of MEF2C protein. **A** The level of HDAC4 and MEF2C protein was remarkably elevated in the cells transfected with corresponding plasmids to overexpress HDAC4 or MEF2C. **B**–**D** The levels of miR-106a-5p, pre-miR-106a, and pri-miR-106a detected by qPCR were significantly increased by MEF2C overexpression. **E** The level of MEF2C protein presented a decreased trend in N2a cells with HDAC4 overexpression. **F** The overexpression of HDAC4 protein inhibited the expression of miR-106a-5p in N2a cells revealed by qPCR. **G** In comparison to the firefly luciferase reporter containing miR-106a promoter, the relative luciferase activity of miR-106a promoter (PM) was elevated by co-transfection with MEF2C overexpression plasmids, and decreased by co-transfection with HDAC4 overexpression plasmids in 293T cells. **H** CUT&TAG qPCR unveiled that MEF2C mainly binds to the promoter of miR-106a at the binding site-2 (BS-2), BS-3, and BS-4 in N2a cells (control vs negative control). Furthermore, elevated MEF2C protein induced by circAKT3 overexpression significantly enhanced the level of binding to the miR-106a promoter at BS-2, BS-3, and BS-4. For H in the figure, ## means Negative control vs Control or CircAKT3 OE group and *P* < 0.01, ### *P* < 0.001, #### *P* < 0.0001, ** (means Control vs CircAKT3 OE group) *P* < 0.01, *** *P* < 0.001, **** *P* < 0.0001. The *P* values were determined by one-way ANOVA with multiple comparison tests (**G**, **H**) or two-tailed unpaired Student’s *t* test (**A**–**F**); * *P* < 0.05, ** *P* < 0.01, *** *P* < 0.001, **** *P* < 0.0001, ns means *P* > 0.05

For our assumption, HDAC4 and MEF2C overexpression was performed with pcDNA3.1 vector plasmids in N2a cells (Fig. 8A). Notably, raising the expression of MEF2C significantly increased the level of miR-106a-5p (Fig. 8B). In order to explore the reason of increased mature miR-106a-5p, the levels of pri-miR-106a and pre-miR-106a after MEF2C overexpression were detected and presented a notable increase (Fig. 8C, D). Moreover, overexpression of HDAC4 induced a decreased trend of MEF2C protein and significantly suppressed the transcriptional activation of miR-106a-5p (Fig. 8E, F).

To identify potential binding sites of MEF2C in the promoter region of miR-106a-5p (upstream 2 kb of transcription start sites), we utilized the Animal TF 3.0 database and Jaspar [21, 22]. Subsequently, we co-transfected luciferase reporter plasmids containing 2 kb fragments of the miR-106a-5p promoter with pcDNA3.1 plasmids to overexpress MEF2C or HDAC4 in 293 T cells. Our findings revealed that the luciferase activities of promoters were significantly enhanced by MEF2C but inhibited by HDAC4 (Fig. 8G). To further confirm the transcriptional activation by MEF2C, CUT&TAG qPCR was performed, indicating that MEF2C predominantly interacts with the promoter of miR-106a-5p at binding sites 2 (BS-2), BS-3 and BS-4 (Fig. 8H). Additionally, N2a cells with circAKT3 overexpression exhibited a higher level of MEF2C-promoter binding in comparison to control cells, indicating the effect of circAKT3 on MEF2C and MEF2Con the miR-106a-5p promoter (Fig. 8H). In conclusion, MEF2C acts as a transcriptional activator of miR-106a-5p, thus forming a feedback loop in the circAKT3/miR-106a-5p/HDAC4/MEF2C pathway.

### Associations between POCD and preoperative neurocognitive disorders revealed by RNA sequencing

Except for POCD, perioperative neurocognitive disorders also conclude preoperative cognitive disorders (CD) [23]. In order to discover the similarities and differences between preoperative CD and POCD, we conducted the whole transcriptome sequencing of the hippocampus from aged male mice, comparing the CD group to the non-cognitive disorders (NCD) group. Remarkably, the expression profiles revealed that miR-106a-5p and MEF2C were also decreased in the CD group in comparison to the NCD group (Fig. S6A, B). Although there is no significant difference in circAKT3 expression between CD and NCD mice (Fig. S6C), we deduced that subtle alterations in upstream circRNAs may regulate downstream miRNAs and mRNAs with fold changes. The shared modified genes in both CD and POCD may be the potential targets responsible for postoperative long-term cognitive decline.

In addition, we conducted a comparison between mice that did not undergo anesthesia and surgery (which includes the CD and NCD groups), and mice that did undergo surgery accompanied by anesthesia, including both POCD and NPOCD groups. GO enrichment analysis of differentially expressed circRNAs indicated positive regulated neuronal death (Fig. S6D). Thus, it could be inferred that anesthesia and surgery possibly upregulate the neuronal apoptosis, and further the increase in neuronal apoptosis would contribute to the development of POCD and postoperative long-term cognitive decline. KEGG analysis for genes targeted by differentially expressed miRNAs revealed that long-term potentiation and glutamatergic synapses were most significantly enriched (Fig. S6E). Consistently, neuronal excitotoxicity has been confirmed to induce neuronal death after anesthesia [24]. The associated primers and fragments are shown in Table 1. The top 30 most significant differentially expressed genes between control and AS groups were listed in Table 2. These findings provide whole transcriptome profiles and multivariate molecular interactions at the transcriptional level for future studies.

**Table 1:** The sequence of primers or templates for qPCR or PCR was used in the study

Gene	Primers(5′-3′)
CircAKT3 F	GGATGAAGTGGCACACACTC
CircAKT3 R	TTTTATATATTCTCCTCTCCGCCA
CircAKT3 joint F (PCR)	GACCGTTTGTGTTTTGTGATGG
CircAKT3 joint R (PCR)	ACTGAGAAGTTGTTGAGGGGA
mmu_circ_0013952 F	GCTCTTCCCGGACCTCATTAC
mmu_circ_0013952 R	ATAGGCTTCCGGCCAAAGGA
mmu_circ_0001147 F	CTGCACTAAATCGGCCTCACA
mmu_circ_0001147 R	AGAGTGCCAGGATTGATGGT
mmu_circ_0009043 F	GAGATTGCGCTAAGGCGG
mmu_circ_0009043 R	TGATATCACCAAGGGGCTGGA
mmu-miR-211-5p RT	GTCGTATCCAGTGCAGGGTCCGAGGTATTCGCACTGGATACGACAGGCAA
mmu-miR-211-5p F	CGCGTTCCCTTTGTCATCCT
mmu-miR-211-5p R	AGTGCAGGGTCCGAGGTATT
mmu-miR-466d-3p RT	GTCGTATCCAGTGCAGGGTCCGAGGTATTCGCACTGGATACGACCTATGT
mmu-miR-466d-3p F	CGCGCGTATACATACACGCAC
mmu-miR-466d-3p R	AGTGCAGGGTCCGAGGTATT
mmu-miR-7688-5p RT	GTCGTATCCAGTGCAGGGTCCGAGGTATTCGCACTGGATACGACGCTCAT
mmu-miR-7688-5p F	GCGTAGCTGGGCATGATCTG
mmu-miR-7688-5p R	AGTGCAGGGTCCGAGGTATT
mmu-miR-106a-5p RT	GTCGTATCCAGTGCAGGGTCCGAGGTATTCGCACTGGATACGACCTACCT
mmu-miR-106a-5p F	GCGCAAAGTGCTAACAGTGC
mmu-miR-106a-5p R	AGTGCAGGGTCCGAGGTATT
HDAC4 F	CAATCCCACAGTCTCCGTGT
HDAC4 R	CAGCACCCCACTAAGGTTCA
MEF2C F	ATCCCGATGCAGACGATTCAG
MEF2C R AKT3 F AKT3 R β-Actin F β-Actin R GAPDH F GAPDH R U6 F U6 R	AACAGCACACAATCTTTGCCT ACCGCACACGTTTCTATGGT TGACAACACCTAAGCCCCAC GCCCATCTACGAGGGCTAT ATGTCACGCACGATTTCC AGGTCGGTGTGAACGGATTTG TGTAGACCATGTAGTTGAGGTCA CGCTTCGGCAGCACATATAC TTCACGAATTTGCGTGTCAT
Common sense primer for AKT3 transcripts	GGACTATCTACATTCCGGAAAG
AKT3 transcript 1 F	GGTGAAGACCCTTGGCTGGTC
AKT3 transcript 2 F	GGGTCTAGATTACTTTTTATTATCATTTTTTTTCCAGTTAC
pre-miR-106a	ATGTCAAAGTGCTAACAGTGCAGGTAGCTTTTTGAGTTCTACTGCAGTGCCAGCACTTCTTACAT
pre-miR-106a F	TCAAAGTGCTAACAGTGCAGGT
pre-miR-106a R	GTGCTGGCACTGCAGTAGAAC
pri-miR-106a	AAGAGTTCGTGGAAGACTTCAAGGTTACACTCCTGGAGTATGCCTTGGCCATGTCAAAGTGCTAACAGTGCAGGTAGCTTTTTGAGTTCTACTGCAGTGCCAGCACTTCTTACATTACCATGGTGATTTAATCAGAGGCCGCTGAGTCCCCTGGTTTCTGCATAG
pri-miR-106a F	TTCGTGGAAGACTTCAAGGTT
pri-miR-106a R	TGCTGGCACTGCAGTAGAAC
BS-1 F (CUT&TAG)	CAAATGGATTTTATGGAATAA
BS-1 R (CUT&TAG)	TTCAATGCCGTGGTGAGGGA
BS-2 F (CUT&TAG)	TTAAGTGGGGTCCCTCACCA
BS-2 R (CUT&TAG)	CTACCAACAGTCACCCGGAC
BS-3 F (CUT&TAG)	CACTTTTACAACCCTCCCCCA
BS-3 R (CUT&TAG)	TCGCTTGTGAGAGTTGCATGG
BS-4 F (CUT&TAG)	TGCCCTCCAACAAAGCAGAGA
BS-4 R (CUT&TAG) Spike in F (CUT&TAG) Spike in R (CUT&TAG)	CGCACAGATGCCAATTACCTT GCCTTCTTCCCATTTCTGATCC CACGAATCAGCGGTAAAGGT
CircAKT3 (mmu_circ_0008480)	GAGAATATATAAAAAACTGGAGGCCAAGATACTTCCTTTTGAAGACAGATGGCTCATTCATAGGCTATAAGGAGAAACCTCAAGATGTGGACTTACCTTATCCCCTCAACAACTTCTCAGTGGCAAAATGTCAGTTAATGAAAACAGAACGACCAAAGCCAAATACATTTATTATCAGATGTCTTCAGTGGACCACTGTTATAGAGAGAACATTTCATGTAGATACACCAGAGGAAAGAGAAGAGTGGACGGAAGCTATCCAAGCCGTAGCCGACCGATTGCAGAGGCAAGAGGAGGAGAGGATGAATTGTAGCCCAACCTCACAGATTGATAATATAGGAGAAGAAGAGATGGATGCGTCTACAACCCATCATAAAAGAAAGACGATGAATGATTTTGACTATTTGAAACTACTAGGTAAAGGCACTTTTGGGAAAGTTATTTTGGTTCGAGAGAAGGCAAGTGGAAAATACTATGCTATGAAGATTCTGAAGAAAGAAGTCATTATTGCAAAGGATGAAGTGGCACACACTCTTACTGAAAGCAGAGTACTAAAGAACACCAGACATCCATTTTTAACATCCTTGAAATATTCCTTCCAGACAAAAGACCGTTTGTGTTTTGTGATGGAATATGTTAATGGCGGAGAG
Promoter of miR-106a (2 kb)	GTTTACCTAAAATACCTTATTCGGGGTATTGTTCTGACTTTAGAAGGGAGGGGTGGAGAGGGTTGAAAACTGGGGTGGGGGTGGGAAGACCATGGGAGGATAGTTTGTTTAATGAACTGTTAAAATTTGGAAATTCTTAAGTGGGGTCCCTCACCACGGCATTGAAACCATCCTCTTAATTTCATTTTAGGCAGGCTGCATCTACTTGTACTCATCTCATAGGATATTTTCATCTCTTCCAGCTCTCTTCTGTCTCTTAACCAAGACGCGTAGTCCGGGTGACTGTTGGTAGTGAGGTTTCTTTGGGAAAATATTCCAAAATCGGGGTAATGGGTTTTCTCATTCTGATGCTCAAGGGAATGTGTTTACTCGTTCCCAGCGCTCCCACTGCAGCTGCAGGGCACAATTAATTATGTTAATAATTTGCGAGAAGCGGATGTAACTCCCCAACTCCTCCCCCCCGGGGGGGTGACAAAAGGGGAAGGAGACATCCTGGGGTCCGACCACGTGATATTATCTTCGCGCCACTTTTACAACCCTCCCCCAGGTTTTGCTTGAGCTCATTGAGGTTTTTTTTTTTTTTAAATCCTGCTTTAATACAATGTCTCTTAAAAGAGGGTCTGAGATATGTTTCCACACCTCTCCATCACTAGCTACCTTTAAAGGTCCAAAGTTGGATTGCAGTCCATGCAACTCTCACAAGCGAGATCTGATTGGATATTAAATTTGGGCTTGCATAGTTTCCAGAAAGCATCAAACAGATGCTCTGGCATACGGTGGGAGGTCTGTTTGTGCGGGTGTTTGAAAGTGGAAGGCAGCCCACCTGTAGGAGTAACACACATTGGAGTAACGAGGGTGCTTTGCATCACTGAGACCTGAGCTGAGCCTGCAATTCTGCTTGAGCTCCACCGCTGGCCAGGGAGCAGGAGACAAGTCTTAGGAGTCTTTCAGTCCTTAGTAATCCGCTCGCCTGAACCCCTACTGTGCACCAGATTGAAACTCACGTGGTGCTCTTGTTCCCTTTTGAGCTTTGAACTGCCACAATCACCCAAGGCAACAGTGTTAGGGAGTAAGGGACCTGTCTTCGTTTCTGAGCCCCAGCTCATCCCTTCCAGTGTCCCCTCTCCTGATAGTGCGTGCTCAGTAACTTCAGGTTTATTTGTTATCTGGTCAATCACTCTTTCCACCATCTAACTCACCAAATATCTATCCTCTTGGACCCGTGCCCTCCAACAAAGCAGAGAAAGTATAGTATGGTTATCCTTTTTTGCCCCATAACCTCCTAGTATGCTTAATTTTGTATTGACGCTAAATCATAATTTTAGTTTTGTACTTGGGATGTGCTTTTAAACAGCCTTCTCTCAAATGAGTCTTAGCTGTGGAAGGGGGAAAAGCAAGGTAATTGGCATCTGTGCGTGCACCCAAGGGACATTACCATTCATAATGCCTGCTAGGAAACTGATTTAAAGGGTAAAGGTGGCGTTCAGCAAGTGTGAAACTTCAGCAGTAGCTTCACATGCCAGAACTGAAAACTTTCACATGGGCAGAGTGGATATGCCGGGTATGTATTTCTTCTCTCCTACAGACTTTCGGGACACTTTCAATCTAGCTGTGAGGTAGTGCGCATGCGCGCACTCGTTGGGACGTGGGGACACCTGGGTCCCACAACTGCTTGGGCCTCGTAGCCTATCTCCAGAGGGGGATGGGACAAGCGCTTCAGGAGCCTGACCGGACAAGGTAGTAATGAGTGCGCCATTCTGGGCAGGCGACTGTGGCACTGGTTTAGACTGGCATCCCATGGGTGCTTGGATTAAGGAAGGACTCTGGTGTTGGGGACAGAAGGGCAAGGCTGAGGGAGCGGGTCCTGTGTGTCACTGGGGCACCGTGGGCTTTGGCCTGGGCGGTGTGGGGGTTCCATTTCCTAATTCCTGTGTGGGCCGAGTGGGCGGGGGCGGGCTTTCCCCCCTAGGCTATTAAAGGCGAAGGGCGGGCTTTTCCCA
Binding site-1	**TTACCTAAAATACCT**
Binding site-2	**TTTCATTTTAGGCAG**GCTGCATCTACTTGTACTCATCTCATA**GGATATTTTCATCTC**
Binding site-3	**TTTTTTTTTTAAATCC**
Binding site-4	**GCTTAATTTTGTATT**GACGCTAAA**TCATAATTTTAGTTTTGTACT**
HDAC4 (NM_207225.2)	ATGAGCTCCCAAAGCCATCCAGATGGACTTTCTGGCCGAGACCAGCCTGTGGAGCTGCTGAATCCTGCCCGTGTGAACCA CATGCCCAGCACGGTGGACGTGGCTACAGCGCTGCCTCTGCAAGTGGCCCCTACAGCAGTACCCATGGACCTGCGCTTGG ACCACCAGTTCTCACTGCCCTTGGAACCTGCATTGCGGGAGCAGCAACTGCAGCAGGAACTCCTAGCACTGAAACAGAAG CAGCAGATCCAGCGGCAGATACTCATTGCAGAGTTCCAGCGTCAACATGAGCAGTTGTCCCGACAGCATGAGGCACAGTT GCATGAACATATCAAGCAGCAGCAGGAGATGCTGGCCATGAAGCACCAGCAGGAGCTGCTGGAGCACCAGCGGAAACTGG AGCGGCACCGGCAAGAGCAGGAGCTGGAGAAGCAGCACCGTGAGCAGAAGCTGCAGCAGCTCAAGAACAAGGAGAAGGGC AAAGAGAGTGCTGTGGCGAGCACAGAGGTGAAGATGAAGCTGCAGGAGTTTGTTCTCAACAAGAAGAAGGCTCTAGCCCA CCGGAACCTGAACCACTGCATTTCCAGCGATCCCCGCTACTGGTATGGGAAGACACAGCACAGCTCCCTTGACCAGAGCT CTCCACCCCAGAGTGGGGTGTCAGCCTCCTACAACCACCCCGTCTTGGGAATGTACGACGCCAAAGATGACTTCCCTCTT AGGAAAACAGCTTCTGAACCTAACCTGAAATTACGCTCAAGGCTTAAGCAGAAAGTAGCTGAGAGACGGAGCAGCCCCCT GTTGCGCAGGAAAGATGGCCCTGTGGCCACTGCTCTAAAAAAGCGACCCCTGGATGTTACAGACTCCGCATGCAGCAGCG CCCCTGGCTCCGGTCCCAGCTCTCCAAATAGCAGCTCTGGCAACGTCAGCACTGAGAATGGCATCGCACCCACTGTGCCC AGCGCTCCAGCTGAGACGAGCTTGGCACACAGACTTGTGACTCGAGAAGGCTCAGTCGCCCCACTTCCTCTCTACACGTC ACCATCCTTACCCAACATCACCTTGGGACTTCCTGCCACTGGCCCTGCCGCTGGTGCGGCAGGTCAGCAGGATGCTGAGA GGCTTGCTCTCCCAGCTCTCCAGCAGCGGATCTTGTTCCCTGGGACCCACCTCACCCCGTACCTGAGCACCTCGCCCCTG GAGAGGGACGGTGCAGCAGCTCACAACCCCCTCCTGCAGCACATGGTCCTGCTGGAGCAGCCACCCACCCAGACACCCCT TGTCACAGACTGGTATCTTTCAGGCCTGGGGGCGCTGCCCCTCCACTCACAGTCCCTGGTTGGTGCGGACAGGGTGTCCC CATCCATTCACAAGCTGCGGCAGCACCGCCCTCTGGGGCGCACGCAGTCAGCACCCCTGCCGCAGAACGCACAGGCCCTG CAGCACCTGGTGATCCAGCAGCAGCACCAGCAGTTCCTGGAGAAGCACAAGCAACAGTTCCAGCAGCAGCAGCTGCACCT CAGCAAGATAATCTCCAAACCTAGTGAGCCACCTCGGCAGCCTGAGAGCCACCCAGAGGAGACAGAGGAGGAGCTCCGTG AGCACCAGGCCTTGCTGGATGAGCCCTACCTAGATCGGCTACCTGGGCAGAAGGAGCCCTCCCTGGCTGGTGTGCAGGTG AAGCAGGAGCCCATTGAGAGTGAGGAGGAAGAAGCGGAGGCCACTCGAGAGACAGAGCCCGGCCAGCGCCCAGCCACTGA GCAGGAGCTGCTCTTCAGACAGCAAGCCCTCCTACTGGAGCAGCAGAGGATCCACCAGTTAAGAAACTACCAGGCATCTA TGGAGGCTGCTGGCATCCCTGTGTCATTTGGCAGCCACAGACCTCTGTCTCGGGCACAGTCCTCCCCAGCATCTGCCACC TTCCCCATGTCAGTCCAGGAGCCCCCCACCAAGCCAAGGTTCACCACAGGTCTTGTGTATGACACACTGATGTTGAAGCA TCAGTGCACCTGTGGGAACACCAACAGCCACCCGGAGCATGCTGGGAGGATCCAGAGCATCTGGTCCCGCCTGCAGGAGA CTGGACTCCGTGGCAAGTGTGAGTGCATCCGTGGACGCAAGGCCACATTGGAGGAGCTGCAGACAGTGCACTCGGAGGCC CACACACTCCTCTACGGCACAAATCCTCTCAACAGACAGAAACTGGACAGTAAGAAACTTCTAGGCTCGCTGACCTCAGT GTTCGTCAGGCTTCCTTGTGGTGGTGTTGGGGTGGATAGCGACACCATATGGAATGAGGTGCACTCGTCTGGGGCAGCCC GCCTGGCTGTAGGCTGTGTAGTGGAGCTGGTCTTCAAGGTGGCCACGGGAGAGCTAAAGAATGGCTTTGCTGTGGTTCGT CCCCCAGGACACCATGCCGAGGAGAGCACACCCATGGGTTTCTGCTACTTTAACTCCGTGGCAGTTGCAGCCAAACTTCT CCAGCAGAGGCTGAATGTGAGCAAGATCCTCATTGTAGACTGGGATGTACATCATGGGAATGGGACCCAGCAGGCCTTCT ACAATGACCCCAATGTTCTCTACATGTCCCTGCACCGCTATGACGATGGGAACTTCTTCCCAGGAAGTGGAGCACCAGAT GAGGTGGGCACAGGGCCAGGCGTGGGTTTCAATGTCAACATGGCTTTCACGGGTGGCCTCGAACCCCCCATGGGAGACGC TGAGTACTTGGCAGCCTTCAGAACGGTGGTTATGCCTATCGCAAATGAGTTTGCCCCAGATGTGGTACTGGTGTCATCGG GCTTCGATGCTGTGGAGGGCCACCCCACACCTCTTGGAGGGTACAATCTCTCTGCCAAATGTTTTGGGTACTTGACAAAA CAGCTGATGGGCTTAGCTGGTGGCCGGCTTGTGCTGGCCCTTGAGGGAGGCCATGACCTGACAGCCATCTGTGATGCTTC TGAAGCCTGCGTGTCTGCTCTGCTGGGAAACGAGCTTGAGCCTCTGCCAGAAAAGGTTCTACATCAGAGACCCAATGCCA ATGCTGTCCACTCCATGGAGAAAGTGATGGACATCCACAGCAAGTACTGGCGCTGCCTGCAGCGTCTGTCCTCCACGGTG GGGCACTCTCTGATTGAGGCGCAAAAGTGTGAGAAGGAAGAAGCTGAGACAGTCACCGCCATGGCCTCGCTGTCTGTAGG CGTCAAACCTGCTGAGAAGAGATCTGAGGAGGAGCCCATGGAGGAGGAACCACCACTGTAG
MEF2C (XM_011244492.4)	ATGGGGAGAAAAAAGATTCAGATTACGAGGATAATGGATGAGCGTAACAGACAGGTGACTTTTACGAAGA GGAAATTTGGATTGATGAAGAAGGCTTATGAGCTGAGCGTGCTGTGCGACTGTGAGATTGCACTGATCAT CTTCAACAGCACCAACAAGCTGTTCCAGTACGCCAGCACTGACATGGATAAGGTGTTGCTCAAGTACACC GAGTACAACGAGCCGCACGAGAGCCGGACAAACTCAGACATTGTGGAGACATTGAGAAAGAAGGGCCTCA ATGGCTGTGACAGCCCAGATCCCGATGCAGACGATTCAGTAGGTCACAGCCCTGAGTCTGAGGACAAGTA CAGGAAAATTAACGAAGATATTGATCTAATGATCAGCAGGCAAAGATTGTGTGCTGTTCCACCTCCCAGC TTTGAGATGCCAGTTACCATCCCAGTGTCCAGCCATAACAGTTTGGTGTACAGCAATCCTGTCAGCACAC TGGGAAACCCCAATCTTCTGCCACTGGCCCACCCGTCTCTGCAGAGGAATAGTATGTCTCCTGGTGTAAC ACATAGACCTCCAAGTGCAGGTAACACAGGCGGTCTGATGGGCGGAGATCTGACATCCGGTGCAGGCACC AGCGCAGGGAATGGATACGGCAACCCCCGGAACTCACCAGGCCTGCTGGTCTCACCTGGTAACCTGAACA AGAATATACAAGCCAAATCTCCTCCCCCTATGAATCTAGGAATGAATAATCGTAAGCCAGATCTCCGCGresultsATCCCACCTGGCAGCAAGAACACGATGCCATCAGTGTCTGAGGATGTGGATCTGCTGTTGAATCAA AGGATAAATAACTCCCAGTCGGCTCAGTCATTGGCTACCCCGGTGGTTTCCGTAGCAACTCCTACTTTAC CAGGACAAGGAATGGGAGGATATCCATCAGCCATTTCAACAACATATGGTACTGAGTACTCTCTGAGTAG CGCAGATCTGTCATCTCTGTCTGGCTTCAACACTGCCAGTGCGCTCCACCTCGGCTCTGTAACTGGCTGG CAGCAGCAGCACCTACATAACATGCCGCCATCTGCCCTCAGTCAGTTGGGAGCTTGCACTAGCACTCATT TATCTCAGAGTTCAAATCTCTCCCTGCCTTCTACTCAAAGCCTCAGCATCAAGTCAGAACCTGTTTCTCC TCCTAGAGACCGTACCACCACCCCTTCGAGATACCCACAACACACCACGCGCCACGAGGCGGGGAGGTCT CCTGTTGACAGCTTGAGCAGCTGTAGCAGTTCCTACGATGGGAGCGACCGAGAGGATCACCGGAACGAAT TCCACTCCCCCATTGGACTCACCAGACCTTCGCCGGACGAAAGGGAAAGTCCTTCAGTCAAGCGCATGCG ACTCTCTGAAGGATGGGCAACATGA

**Table 2:** The top 30 most significant differentially expressed genes of the hippocampus between control and AS aged mice (AS vs control group)

Gene_id	Gene	log2Fold change	*p* value
XM_017313664.1	Arpp21	7.540158	2.14E-05
NM_028059.2	Zfp654	5.956425	1.49E-05
XM_006519126.2	Ercc6	5.79488	0.001297
XM_006527454.3	Stambpl1	5.791363	0.002762
XM_006513121.3	Pip4k2c	4.811344	0.003321
NM_001177853.1	Asph	4.796999	0.00026
XM_006514837.3	Fam161a	4.768188	0.003459
NM_007963.2	Mecom	4.5437	0.003257
XM_006531909.3	Mbtd1	4.523288	0.002289
XM_006526325.3	Mpp7	4.467697	0.001734
XM_017315067.1	Atp9a	4.029276	0.001902
XM_017313585.1	Mlip	3.977897	0.000466
XM_006505501.1	Eif2ak3	3.846224	6.94E-06
XM_017313981.1	Ppfia2	3.823266	5.41E-05
NM_030597.3	Lsm2	3.723786	0.002599
XM_017315406.1	Mef2c	− 2.5629	1.17E-07
XM_006530976.3	Def8	− 2.66479	3.69E-05
XM_017314983.1	Nrxn3	− 2.67413	0.00024
XM_006523043.3	Robo2	− 2.84188	0.000631
XM_017320073.1	Ptprd	− 2.88619	5.14E-11
XM_006498029.3	Ppp1r26	− 3.46353	6.97E-05
NM_019778.2	Zbtb20	− 3.7445	6.37E-07
XM_011239240.1	Dgkz	− 3.76674	0.00086
XM_011246278.1	Epb41l3	− 4.06288	0.000253
XM_006497189.1	Dusp10	− 4.14608	0.000522
XM_011243570.1	Ankrd24	− 4.34804	0.000265
XM_017312750.1	Clasp1	− 4.46648	2.54E-05
XM_017312508.1	Mmaa	− 5.11236	9.16E-05
NM_001081218.1	Hcfc2	− 6.25865	0.000189
NM_010663.3	Krt17	− 6.81775	0.000168

## Discussion

Our study reveals the differentially expressed profiles of circRNA, miRNA, and mRNA between NPOCD and POCD groups. This provides additional targets and potential molecular interactions for further preclinical research on POCD. Bioinformatics analysis of DEGs and TUNEL assay revealed an increase of neuronal apoptosis in the hippocampus of POCD mice, which aligns with previous studies [25, 26]. We discovered that circAKT3 was downregulated in the hippocampal neurons of POCD mice. Different from other isoforms of AKT, the host gene AKT3 is mainly expressed in the brain, suggesting its unique role in the central nervous system [27]. Furthermore, circAKT3 is predominantly localized in the cytoplasm of N2a cells and neurons of the hippocampal CA3 region. The CA3 region of the hippocampus is acknowledged to have a crucial function in spatial working memory, and inputs from the left CA3 to the CA1 region result in enhanced long-term potentiation [28]. Consequently, we performed the overexpression of circAKT3 in the neurons of the bilateral hippocampal CA3 region, which mitigated neuronal apoptosis and improved postoperative cognitive performance. This indicates that circAKT3 contributes to hippocampal-dependent memory after anesthesia and surgery.

Furthermore, when comparing POCD with NPOCD and control mice, miR-106a-5p was markedly reduced in POCD mice and was observed to co-localize with circAKT3. This co-localization suggests potential interactions between circAKT3 and miR-106a-5p. Combined bioinformatics analysis with DEGs between NPOCD and POCD, HDAC4 emerged as a candidate target of miR-106a-5p. Through the overexpression of circAKT3 in vitro and in vivo, we discovered that circAKT3 elevates the level of miR-106a-5p and remarkably reduces the expression of the HDAC4 protein. In turn, an inhibitor of miR-106a-5p increases the level of HDAC4 protein, which reverses the effect of circAKT3 overexpression. While this mode of regulation differs from typical ceRNA networks, it has been reported previously [29]. However, the precise mechanism behind this phenomenon has not been elucidated in their study. A recent study proposed that circRNA could promote the processing and mature of miRNA by acting as a scaffold [30]. In this study, actinomycin D treatment validates that circAKT3 enhances the stability of miR-106a-5p. We also exclude the direct binding between circAKT3 and HDAC4 protein through RIP assay. Therefore, based on the high co-localization of circAKT3 and miR-106a-5p and the results of the dual-luciferase assay, we hypothesize another modulation that may occur through different binding sites or common protein platforms with miR-106a-5p for their secondary structures. Previous research has shown that a reduction in miR-106a-5p was associated with increased amyloid beta deposition [31, 32]. Moreover, overexpression of miR-106a-5p was reported to notably reduce the level of pro-inflammatory cytokines, such as interleukin-1β, interleukin-6, and tumor necrosis factor-α in LPS-stimulated inflammation [33]. Additionally, HDAC4, as a class II histone deacetylase, binds with and inhibits the activity of MEF2C [17]. Transcriptional changes in neurons in Alzheimer's disease (AD) are specifically regulated by MEF2C, without the involvement of other members of the MEF2 family [34]. The DEGs of NCD and CD-aged mice in our database also shed light on the role of miR-106a-5p and MEF2C. Decreased transcriptional activation of MEF2C contributes to neuronal apoptosis and impairs synaptic plasticity and memory [8, 20, 34]. The overlap between the altered genes in preoperative CD and POCD further demonstrates that MEF2C plays an indispensable role in mitigating perioperative neurocognitive disorders in the elderly.

What’s more, our study also found that MEF2C promoted the transcription of miR-106a-5p by activating its promoter. Similarly, previous research has reported that MEF2C activates the transcription of miR-133a in neurons [8]. However, it remains unknown whether MEF2C promotes the transcription of circRNA. In terms of glia-neuron crosstalk, astrocytic apolipoprotein can transport miRNAs that silence pivotal enzymes involved in cholesterol synthesis and promote histone acetylation in neurons [35]. Moreover, microglia activated by tau also inhibit the neuronal transcription networks controlled by MEF2C [34]. Inhibition of MEF2C in neurons leads to decreased transcription activation and subsequently results in astrocyte activation [8]. These studies shed light on additional functions and potential mechanisms of non-coding RNAs.

However, there are also some limitations in our study. Firstly, the profiles of differentially expressed genes from transcriptome presented little overlap with other transcriptome studies of POCD [36, 37], due to the limitation of sample size, different animal ages, and models. The coincidence of POCD and AD predominantly reveals the targets involved in postoperative long-term cognitive decline. Additionally, MEF2C was validated to promote the transcriptional activation of miR-106a promoter. We did not investigate whether MEF2C promotes the transcriptional activation of circAKT3. Moreover, owing to the lack of commercially available antibodies, we only detect the effect of circAKT3 overexpression on two transcripts of AKT3 without further detecting the isoforms of AKT3 protein. Clinical samples for transcriptome sequencing are also deficient.

**Fig. 9:**
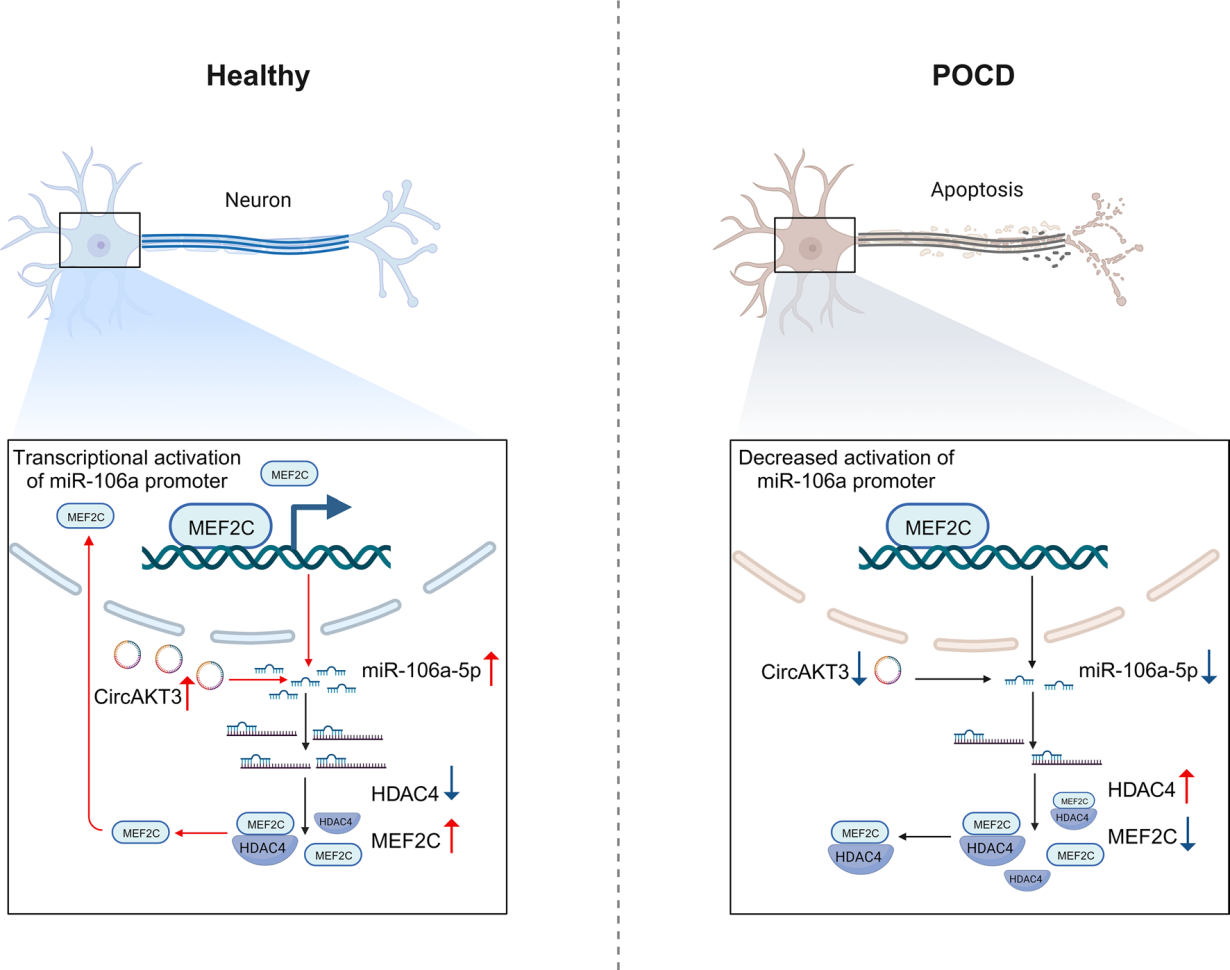
CircAKT3 improved postoperative cognitive dysfunction by stabilizing the feedback cycle of the miR-106a-5p/HDAC4/MEF2C axis. CircAKT3 could elevate the level of miR-106a-5p, which further decreased the level of HDAC4 protein and increased the level of MEF2C protein to inhibit neuronal apoptosis. MEF2C would in turn promote the transcriptional activation of miR-106a and form a feedback loop. However, circAKT3 is reduced in the hippocampal neuron of POCD mice and increased the expression of HDAC4 protein through a decrease in miR-106a-5p. Then the effect of MEF2C on miR-106a promoter and neuronal apoptosis was weakened, which promoted the occurrence and development of POCD. The schematic picture was drawn by BioRender

Collectively, whole transcriptome sequencing of the hippocampus in aged mice was performed to disclose the significantly changed circRNA, miRNA, and mRNA between POCD and NPOCD groups. Specifically, we found that circAKT3 reduces the levels of HDAC4 protein by increasing the expression of miR-106a-5p in the neuron of the hippocampus, which ultimately enhances the activity of MEF2C. Furthermore, we discovered that MEF2C promotes the expression of miR-106a-5p by binding to its promoter, thus creating a feedback loop of regulation (Fig. 9). Compared to sequencing circRNAs or miRNAs alone, conducting whole transcriptome sequencing and analysis provides additional insights into RNA interactions and regulation. In addition, it is worth noting that the formation of the ceRNA network heavily relies on the stoichiometric relationships of circRNAs/miRNAs. The interaction and regulation of RNAs and proteins are remarkably complex. Given the non-classical regulation observed among non-coding RNAs, it is imperative to consider various possibilities and directions to unravel the role of non-coding RNAs in multiple pathophysiological conditions.

## Materials and methods

### Animals

All mice were obtained from the Laboratory Animal Centre of Tongji Hospital, Huazhong University of Science and Technology. Aged male C57BL/6 mice (18 months, 33–37 g) were housed in the Animal Center of Tongji Hospital (12 h light/dark cycle, 23 ± 2 ℃, food and water ad libitum, standard specific pathogen-free level). In addition, the whole study was approved and supervised by the Experimental Animal Ethics Committee of Affiliated Tongji Hospital (TJH-201801011), Tongji Medical College, Huazhong University of Science and Technology (China).

### Surgery and anesthesia model

Aged mice were randomly allocated into two groups. The anesthesia and surgery (AS) group accepted open tibial fracture along with intramedullary fixation surgery under inhalation anesthesia using isoflurane as previously reported [38]. In brief, mice were kept in a chamber filled with 100% oxygen and 3% isoflurane. After Loss of Consciousness, the skin under the knee of the mice was incised and a sterile pin of about 300 um was inserted into the cavity of tibial marrow with 1.5% maintained isoflurane anesthesia. Then tibia was fractured at the midpoint and the skin was closed with 4–0 resorbable threads. Anesthesia and surgery were performed with sterile instruments and lasted 30 min for each mouse. No treatment was made on the control group. Further, mice who underwent anesthesia and surgery were divided into non-POCD (NPOCD) and POCD groups.

### Behavioral tests

#### Open field test

The open field test (OFT) was used to detect locomotor activity on the seventh day after anesthesia and surgery. Mice were put in an open white box measuring 40 cm × 40 cm × 40 cm and were given 5 min to freely explore their surroundings. The box was cleaned with 75% ethanol to eliminate the effects of odor. Total distance (cm) and average speed (cm/s) of movement were tracked and analyzed by the video tracking system (Zhongshi Technology, China).

#### Morris water maze test

To assess spatial learning and memory, the Morris water maze (MWM) test was conducted on the 8th day following anesthesia and surgery. MWMT consists of five consecutive daily training trials and a spatial probe trial on the sixth day. The Morris water maze test was conducted in a circular pool with a diameter of 120 cm and a depth of 50 cm. The pool with water was partitioned into four quadrants. The water temperature was kept at 23 ± 1 ℃. In the target quadrant, a round platform with a diameter of 10 cm was placed and submerged 1 cm below the surface of the water. Mice were put at a fixed starting point within each quadrant and allowed for 60 s to locate the fixed platform. After a successful location, mice would stay 15 s there. If the mice were unable to find the platform, they would be led to and permitted to remain on the platform for 15 s. This training procedure continued for a total of 5 days, during which the escape latency (time spent to land the hidden platform) was automatically recorded and analyzed by the video tracking system (Zhongshi Technology, China). Next, on the 13th day after surgery, the platform was removed from the pool to conduct a probe test. From each quadrant, mice were put into the water pool and given 60 s to freely explore. The number of times the mice crossed the previous location of the platform and the residence time spent exploring the target quadrant were recorded in order to evaluate their memory retention ability.

#### Fear condition test

Fear condition test was conducted as described in the report previously [39]. In brief, mice were put into the chamber for 120 s adaptation on the first day. Then mice were given sound stimulation (75db, 30 s), during which the electronic shock was given at 28-30 s. Two stimulations stopped simultaneously and were repeated for three cycles. The interval time between cycles was 120 s. After that, the mice stayed in the chamber for 120 s. Ethanol (70%) was used to clean the chamber and eliminate the interference of animal odor. In the test session (24 h after the training session), mice were put into the same chamber and no stimulation was given. The freezing time during the 120 s was recorded for assessing the hippocampus-independent memory. After 2 h, the environment of the chamber was changed and mice were given 120 s for adaptation. The stimulation of sound was then given and the freezing time of mice was recorded to evaluate the hippocampus-dependent memory.

### Whole transcriptome sequencing

#### RNA isolation and library preparation

Total RNA extracted by TRIzol reagent (Invitrogen, USA) was performed. The purity and quantification of RNA were evaluated by the spectrophotometer (NanoDrop 2000, Thermo Scientific, USA). RNA integrity was detected by the Bioanalyzer (Agilent 2100, Santa Clara, USA). The samples with qualified purity, quantity, and integrity were used for subsequent library construction. After removing the ribosomal RNA, we constructed the libraries using the VAHTS Universal V6 RNA-seq Library Prep Kit in accordance with the guidelines. Then OE Biotech (China) conducted the transcriptome sequencing and analysis of expressed gene profiles.

#### RNA sequencing analysis process

The libraries underwent sequencing using an Illumina Novaseq 6000 platform, which generated 150 bp paired-end reads. Fastq was used as an initial step to preprocess the raw reads in fastq format for each sample, eliminating reads with low quality [40]. HISAT2 was employed to align the cleaned reads to the reference genome after the removal of low-quality reads [41]. The gene read counts were acquired through the utilization of HTSeq-count [42] and then FPKM [43] of each gene was calculated. To assess the biological replication of samples, PCA (Principal Component Analysis) analyses were conducted.

After the differential expression analysis with DESeq2 [44], DEGs were determined based on the threshold of fold change (FC) < 0.5 or > 2 and the p value < 0.05. To illustrate the expression patterns of genes across various groups, a hierarchical cluster analysis of DEGs was conducted using R (v 3.2.0). To visualize the profiles of up-regulated or down-regulated DEGs, A radar map depicting the expression levels of the top 30 genes was generated. Subsequently, the GO [45], KEGG [46], Reactome, and Wiki Pathways enrichment analyses of DEGs were conducted to identify the significantly enriched term. R was utilized to generate column diagrams, chord diagrams, and bubble diagrams for visualizing the significant enrichment term. GSEA software was utilized to perform the Gene Set Enrichment Analysis (GSEA) [47, 48].

#### CircRNA analysis

Find_circ (v 1.2) and CIRI2 [49] software were used to identify the circRNAs in the library. The parent gene of circRNA was annotated according to its genomic position. The Circbase and CIRCpedia were used to identify the known circRNAs. Junction reads per billion mapped reads (RPB) were utilized to quantify circRNA, and DEGseq [50] was used to calculate the differential expression profiles of circRNA. The p value was calculated by negative binomial distribution test (NB). The threshold of P value and FC for significantly differentially expressed circRNAs (DECs) were set as described above. Based on the hypergeometric distribution, GO [45], KEGG [46] pathway, Reactome, and Wiki Pathways enrichment analyses of the differential expression of circRNA's parent genes were performed to evaluate the function of circRNA's parent genes. Pearson's coefficient was used to calculate the expression correlation between DECs and DEGs. The significantly related circRNA-gene pairs were screened with the criteria of p < 0.05 and |cor|> 0.8. GO, KEGG Pathway, Reactome, and Wiki Pathways enrichment analysis were performed for genes significantly co-expressed with circRNAs to predict the function of circRNAs. The binding sites of circRNAs and miRNAs were predicted by miRanda (v 3.3a) software. CircRNA-RNA binding protein pairs were obtained based on the starbase database.

#### Small RNA sequencing experimental method

Total RNA was extracted by the mirVana miRNA Isolation Kit (Ambion) following the protocol of instruction. The quantitation of total RNA and evaluation of RNA integrity were conducted as previously described. Total RNA (1000 ng) extracted from each sample was utilized to construct the small RNA libraries with NEBNext Small RNA Library Prep Set for Illumina kit (NEB, USA). In brief, the adapter-ligated RNA underwent reverse transcription to generate cDNA and subsequent amplification with PCR. The products of PCR (140–160 base pairs in length) were isolated to create the miRNA libraries. Next, the libraries were assessed and performed sequencing with the Illumina Novaseq 6000 platform.

#### Small RNA sequencing analysis process

The raw reads were generated by base calling that converted the basic reads into sequence data. Subsequently, reads with low quality were filtered out along with those containing 5' primer contaminants and poly (A) sequences. Also, we eliminated reads lacking the 3' adapter and insert tag, as well as reads shorter than 15 nucleotides (nt) or longer than 41 nt from the raw data. The length distribution of the clean sequences in the reference genome was determined, and then the sequences were aligned and subjected to the Bowtie [51] search against Rfam v.10.1 [52], rRNA, scRNA, Cis-reg, snRNA, tRNA, and other RNAs were annotated and filtered. The cDNA sequence and Repbase database of corresponding species repeat sequences were identified with Bowtie software. We also identified the mature miRNAs by aligning against the miRBase v22 database [53], and then analyzed the expression patterns of different samples. Subsequently, unannotated reads were subjected to analysis by miRDeep2 for the prediction of novel miRNAs [54]. Subsequently, unannotated reads were subjected to analysis by miRDeep2 for the prediction of novel miRNAs. Differential expression of miRNAs was calculated and filtered using a threshold of p value < 0.05 and a fold change (FC) greater than 2 or less than 0.5. The targets of differentially expressed miRNAs were identified with miRanda [16]. The following parameter settings were employed: S ≥ 150, ΔG ≤ -30 kcal/mol, and strict 5' seed pairing requirement. Finally, the GO and KEGG enrichment analysis of different expressed miRNA-target-Gene was conducted.

### RNA extraction and quantitative real-time polymerase chain reaction (qRT-PCR)

The total RNA of the hippocampus was extracted according to the manufacturer’s instructions for the RNAiso Plus kit (Takara). The purity and concentration of total RNA were detected by Nanodrop ND-1000 spectrophotometer (NanoDrop Technologies, Wilmington, DE, USA). RNAs with high purity (1000 ng) were reverse-transcribed using the HiScript® III 1st Strand cDNA Synthesis Kit (+ gDNA wiper) and miRNA 1st Strand cDNA Synthesis Kit (by stem-loop) (Vazyme Biotechnology, China), respectively. Besides, RNA was treated with RNaseR (EPicentre, USA) to digest linear RNA (37 °C, 15 min) before the reverse transcription polymerase reaction of circRNA. All qPCR was performed on an Applied Biosystems StepOnePlus Real-Time PCR System (Applied Biosystems, USA) using the SYBR^®^ qPCR Master Mix (Vazyme Biotechnology, China). Glyceraldehyde 3-phosphate dehydrogenase (GAPDH), U6, and β-Actin were used as endogenous controls respectively. The reaction conditions for qPCR were set up based on the manufacturer’s protocol: incubation was set at 95 °C for 2 min, followed by 45 cycles of 30 s at 95 °C, 30 s at 68 °C, and 10 s at 72 °C. Expressional levels were quantified using the 2^−ΔΔCT^ method. The sequences of all primers are listed in Table 1.

### RT PCR and agarose gel electrophoresis

RT PCR for AKT3 transcripts was performed as previously reported [15]. Total RNA (2 μg) extracted from the hippocampi of aged mice was reverse transcribed to cDNA using the Hifair® III 1st strand cDNA synthesis kit containing gDNA digester plus (Yeasen, China). The hippocampi of mice were injected with the recombinant adeno-associated virus to overexpress circAKT3 (rAAV-hSyn-mmu-circAKT3-nEF1α-EGFP) or the empty vector (rAAV-hSyn-EGFP) to exclude the interference of rAAV injections. Next, cDNA was amplified using 2 × Phanta^®^ Max Master Mix with Dye Plus (Vazyme, China) and common sense primer (5′-GGACTATCTACATTCCGGAAAG-3′) with AKT3 transcript 1 primer (5′-GGTGAAGACCCTTGGCTGGTC-3′) or AKT3 transcript 2 primer (5′-GGGTCTAGATTACTTTTTATTATCATTTTTTTTCCAGTTAC-3′). The primer sequences were synthesized as reported previously [15]. And the PCR protocol consisted of preliminary denaturation (95 ℃, 3 min), repeated 35 cycles containing denaturation (95 ℃, 15 s), annealing (54 ℃, 15 s) and extension (72 ℃, 30 s), and a further extension at 72 ℃ for 5 min. Afterwards, the Semiquantitative RT-PCR was scored by agarose gel electrophoresis (1%) mixed with GelRed nucleic acid dye (Yeasen, China) and photographed by the ultraviolet imaging system.

### Western blotting assay

Whole tissue proteins from the hippocampus were homogenized using the pre-cold radioimmunoprecipitation (1 ×) assay buffer (Boster, China) mixed with inhibitors of protease and phosphatase (Boster, China). And lysates were further depolymerized with an ultrasonic cell disrupter (Scientz, Ningbo, China). The homogenized lysates were centrifuged (12,000 g, 15 min, 4 ℃) and the resulting supernatant was kept. Concentrations of total protein were detected according to the instructions of the bicinchoninic acid protein assay kit (Boster, China). Next, Supernatants were mixed with 5 × protein loading buffer from Boster (China) and boiled for 5 min.

Following this, hippocampal protein lysates of protein (30 μg) were loaded onto 10% or 12% sodium dodecyl sulfate–polyacrylamide gels for electrophoresis. The separated proteins were then transferred to 0.45 μm polyvinylidene fluoride membranes (Millipore, Bedford, MA, USA). The membranes were blocked using a solution of 5% bovine serum albumin (Biofroxx, German) dissolved in Tris-buffered saline (Servicebio, China) with 0.1% Tween 20 (TBST). The blocking step was carried out at room temperature for 1 h. Then membranes were incubated with corresponding primary antibodies including anti-HDAC4 (1:1000; Affinity, China), anti-MEF2C (1:1000; Proteintech, China), anti-β Actin (1:3500; ABclonal, China), anti-Bcl2 (1:1000; Proteintech, China), anti-Bax (1:1000; Cell Signaling Tech, USA), anti-cleaved caspase 3 (1:1000; Cell Signaling Tech, USA) and anti-AKT3(1:1000; ABclonal, China) overnight at 4 ℃. The next day, stripes were washed with TBST (3 times, 10 min each time) and incubated with goat anti-rabbit or goat anti-mouse IgG horseradish peroxidase antibody (1:5000, proteintech, China) at room temperature (1 h). Subsequently, stripes were also washed three times for 10 min within TBST. Finally, protein bands were exposed with enhanced chemiluminescence (Abbkine Scientific, China), and photographed using the ChemiDoc XRS chemiluminescence imaging system (Bio-Rad, Hercules, USA).

### Immunofluorescence staining and Fluorescence in situ hybridization

Immunofluorescence (IF) staining was conducted to identify the cellular localization and expression levels of proteins in N2a cells and the hippocampus, following a previously established protocol [55]. Briefly, the entire brain of mice was fixed in 4% paraformaldehyde (PFA) for 48–72 h after perfusion with phosphate-buffered solution (PBS) and cut into paraffin Sects. (4um thickness). After deparaffinization and hydration procedures, antigen of sections were repaired through water-bath of sodium citrate buffer (Servicebio, China) and heated by the microwave (2 times, 7 min each time). Then sections immersed in antigen repair buffer were left outside for natural cool down. After that, sections were washed with PBST (3 times, 10 min per time) and permeabilized with 0.25% TritonX-100 (Servicebio, China) for 15 min. Sections were blocked with goat serum (Boster, China) for 1 h at 26 ℃. Next, sections were incubated with primary antibodies including anti-HDAC4 (1:100; Abclonal, China), anti-MEF2C (1:100; Abclonal, China), and anti-neuronal nuclei antibody (1:100; Abcam, UK) at 4 ℃ for 14 h. On the next day, sections were kept shielded from light after washing for 30 min (10 min, 3 times) with PBST and left to incubate with a mixture of secondary antibodies consisting of Alexa 488-conjugated goat anti-mouse (1:150; Abbkine Scientific, China) and 594-conjugated goat anti-rabbit antibody (1:150; Abbkine Scientific, China) at 26 ℃ for 1 h. Followed by 3 washes with PBST, 4, 6-diamidino-2-phenylindole (DAPI; Abbkine Scientific, China) was incubated for 9 min at 26 ℃. After the antifluorescent quenching seal, mages were captured using a fluorescence microscope (Olympus BX51, Japan). Fluorescence in situ hybridization (FISH) of cells was performed according to the protocols of the Fluorescent in situ hybridization kit (Ribobio, China). MiR-106a-5p probe was synthesized by Ribobio (Guangzhou, China), and the circAKT3 probe was synthesized by Calm Biotechnology (Shanghai, China). Cell climbing slices were firstly fixed with 4% PFA for 20 min and permeabilized using 0.3% Triton X-100 for 15 min. Then slices were prehybridized for 30 min and hybridized using probes of circRNA and miRNA for 16 h at 37℃. The following day cell climbing slices were washed sequentially with 4 × ssc (5 min, 3 times), 2 × ssc (5 min, 1 time), and PBS (5 min, 1 time). After this, cells or slices were incubated within DAPI for 9 min and washed in PBS for 5 min. Finally, the cell climbing slices were taken out and sealed before being photographed.

Moreover, FISH of the hippocampus was performed as previously reported. After the fixation with perfusion, collected entire brains were postfixed in 4% PFA overnight and dehydrated using 20% (w/v) as well as 30% sucrose dissolved within PFA for 24 h at 4 °C. Afterward, the brain was cut into slices containing the hippocampus (10 um thickness) using a microtome (Leica RM2255 microtome). Slices were blocked with goat serum at 37 ℃ for 1 h after permeabilization and then hybridized using probes overnight at 37 ℃. After washing as described above, hippocampal slices were incubated with corresponding primary antibodies in antibody diluent solution for 14 h at 4 °C. Then slices were washed 3 times (10 min one time) and incubated with secondary antibodies for 1 h at 26 °C. Finally, slices were incubated with DAPI after washing and sealed. All brain slices were photographed with the fluorescence microscope (Olympus BX51, Japan).

### TUNEL staining

TUNEL staining was performed according to the protocol reported [56]. Hippocampal paraffin sections were subjected to dewaxing and hydration and digested using protease K (15 min, 37 °C). Afterward, sections of the hippocampus were washed in PBS for 15 min and stained by a TUNEL cell apoptosis detection kit (Beyotime, China). After washing and sealing, hippocampal sections were photographed using the fluorescence microscope (Olympus BX51, Japan).

### Stereotaxic injection of recombinant adeno-associated virus

The stereotactic injections of recombinant adeno-associated virus in hippocampal CA3 were performed as reported [8]. Recombinant AAV vectors for circAKT3 overexpression (rAAV-hSyn-mmu-circAKT3-nEF1α-EGFP) or empty virus vectors (rAAV-hSyn-EGFP) to exclude the interference of viral injections were obtained from BrainVTA (BrainVTA, China). After anesthesia, mice were head-fixed and two holes of the skull were generated above the hippocampal CA3 region. Recombinant AAVs (1025 TU/ml, 120 nl) were stereotaxically injected (80 nl/min) into the CA3 region of the hippocampus (anterior/posterior, − 2.1 mm; medial/lateral, ± 2.6 mm; dorsal/ventral, − 2.4 mm) using a NanoFil needle (WPI, USA). The needle after injection was kept for 10 min and withdrawn with a slow velocity. Next, the skin was sutured with 4–0 lines. All the mice were kept on a heating pad to assist in their recovery process.

### Cell culture

Murine neuroblastoma Neuro-2a (N2a) cells and Human Embryonic kidney 293 T cells were maintained with the complete medium. The complete medium was composed of Dulbecco’s modified Eagle’s medium (Invitrogen, Carlsbad, CA), fetal bovine serum (10%; Invitrogen, CA), and mixed penicillin/streptomycin (1%; Invitrogen, CA). Cultures of primary neurons were isolated from embryonic day 18 C57 fetal mice as reported previously [57]. The cortex and hippocampus of embryos were dissected and digested in 0.05% pancreatic enzymes for 15 min at 37 °C. After neutralization and centrifugation, dissociated neurons were evenly plated onto the glass coverslips in a 24-well or 12-well plate. The sterile round coverslips were pre-coated with poly-D-lysine (Sigma, USA) for 12 h. Moreover, neurons were firstly cultured in the plating medium (10% fetal bovine serum and 1% penicillin/streptomycin in DMEM/F12 medium) for 4–6 h then replaced with maintenance medium (2% B-27, 1% glutamine in Neurobasal plus medium) after adhesion. Half media was then changed every 3 days. All mediums and supplements for primary neurons were purchased from Invitrogen (USA). All cells were cultured in an incubator (37 ℃, 5% CO_2_).

### Lentivirus, miRNA mimics, and plasmid transfection

The lentivirus-based vectors for circAKT3 overexpression (LV-CMV-mmu-circAKT3-nEF1α-EGFP) and its negative control (LV-CMV-EF1α-EGFP) were packaged by BrainVTA (China). The overexpression plasmid of pcDNA3.1 containing HDAC4 or MEF2C, miR-106a–5p mimics, negative control, and inhibitor were all synthesized by Ribobio (China). Lentivirus transfection for N2a cells was performed as previously reported [58]. Transfection of miRNA mimics, inhibitors, and plasmid in N2a cells was carried out using Lipo3000 transfection reagent (Invitrogen) following the instructions. The efficacy and effect of lentivirus and plasmid transfection were detected by qPCR and western blot.

### Actinomycin D treatment

The treatment was performed as previously described [59]. In brief, N2a cells (control and circAKT3 overexpression group) were treated with Actinomycin D (10 μg/ml; MCE, China) and harvested at 0, 2, 4, 6, and 12 h. Total RNA was extracted and miR-106a-5p was detected using miRNA 1st Strand cDNA Synthesis Kit (by stem-loop) (Vazyme Biotechnology, China) and SYBR® qPCR Master Mix (Vazyme Biotechnology, China). Relative expression of miR-106a-5p in two groups at different time points was compared to the expression of miR-106a-5p in the control group at 0 h and U6 was used as an internal reference.

### RNA immunoprecipitation assay

RNA immunoprecipitation assay was conducted according to the instructions of RNA immunoprecipitation kit (GENESEED, China). N2a cells (1 × 10^7^ cells/sample) were lysed after adding protease inhibitor and RNAase inhibitor and divided into three groups (input, anti-IgG, and anti-HDAC4 group). Then the samples were incubated with primary antibody anti-IgG (Proteintech, China) or anti-HDAC4 (Cell Signaling Technology, China) for antigen capture overnight at 4℃. The samples were next washed five times. RNA or proteins were extracted from the sample and purified for qPCR and Western blotting. The relative expression of circAKT3 was calculated by the formula (2^−△△Ct^). 2^−△△Ct^ = (Ct_IP_-Ct_Input_)-(Ct_IgG_-Ct_Input_) = X. Then the relative expression level of circAKT3 in the IP group compared to the IgG group was 2^−X^ fold.

### Cell counting kit-8 assay

N2a cells in the control and circAKT3 overexpression group were evenly seeded in a 96-well plate (5000 cells per well, three replicates) and treated with LPS (100 ng/ml) for 12 h. Cell viability at 0, 24, 48, and 72 h after changing the medium was evaluated by Cell Counting Kit-8 assay (MCE, USA) according to the instructions. Next, the cells were incubated with an assay for 40 min (37℃). Then the absorbance was detected at 450 nm by a microtiter plate reader (Multiskan FC, ThermoScientific, USA).

### CUT&TAG qPCR

CUT&TAG assay was performed following the instructions of the CUT&TAG kit (Vazyme Biotechnology, Nanjing, China). One hundred thousand cells per condition were counted and used to bind the Concanavalin A-attached magnetic beads (treated with binding buffer). Digitonin was used to permeabilize cell membranes and magnetic beads were incubated with MEF2C antibody (1:50; Proteintech, China) for 16 h at 4 ℃. Next, the magnetic beads with cells were incubated with an anti-rabbit secondary antibody (1 h, at 23 ℃) and then incubated with protein A/G (fused with transposons, 1 h, room temperature). Subsequently, DNA segments cleaved by transposons were extracted and released to perform a qPCR assay. DNA spike was added as the internal reference and the sequence for qPCR was listed in Table 1.

### Dual-luciferase reporter assay

Dual luciferase reporter assays were conducted as the previous report described [60]. The luciferase reporter plasmids including circAKT3-wild type (wt), circAKT3-mutant type (mut), HDAC4-wt, HDAC4-mut, and promoter of miR-106a-2 kb were constructed by Ribobio (Guangzhou, China). The 293 T cells were implanted in 24-well plates (0.5 × 10^5^ per well). Four hours later, cells were transfected with luciferase reporter plasmids (0.8 ug), and Renilla luciferase (0.1 ug) and performed miRNA mimics, inhibitors, or plasmids of HDAC4 or MEF2C for 6 h. Finally, cells were lysed at 48 h after transfection and detected the luciferase activities using a Dual-Luciferase Reporter Assay kit (Vazyme Biotechnology, China). Relative luciferase activity was calculated as the ratio of firefly to Renilla luciferase activity.

### Statistical analysis

Results are presented as mean ± SD in the article and independent animal or cell experiments were performed at least three times. All analysis for statistics were analyzed using GraphPad Prism 9.5 or the SPSS 18.0 software. Two-tailed Student’s t-test was used to detect the comparisons of two independent groups. Moreover, one-way ANOVA with multiple comparisons followed by post hoc tests was used to evaluate the statistical significance among different groups. Two-way ANOVA with repeated measures was used to analyze the differences in escape latency among different groups. The evaluation criteria of significance for all the statistical tests is a P value less than 0.05.

### Supplementary Information

Below is the link to the electronic supplementary material.

Supplementary file1 (DOCX 34467 KB)

## Data Availability

This article and supplementary files conclude all the data used in this study. The data is available for reasonable request.
