# Supplementary material for: CircAKT3 alleviates postoperative cognitive dysfunction by stabilizing the feedback cycle of miR-106a-5p/HDAC4/MEF2C axis in hippocampi of aged mice
Source: Cell Mol Life Sci. 2024 Mar 13;81(1):138. doi: 10.1007/s00018-024-05156-9 (PMC10937803; doi:10.1007/s00018-024-05156-9)
Supplement: Supplementary file 1 — Supplementary file1 (DOCX 34467 KB) [file 18_2024_5156_MOESM1_ESM.docx]

CircAKT3 alleviates postoperative cognitive dysfunction by stabilizing the feedback cycle of miR-106a-5p/HDAC4/MEF2C axis in hippocampi of aged mice

Xuan Wang^1^, Xiaole Tang^2^, Pengfei Zhu^1^, Dongyu Hua^1^, Zheng Xie^1^, Mingke Guo^1^, Mengxin Que^1^, Jing Yan^1^, Xing Li^1^, Qian Xia^1^, Xiaoxiao Luo^3^, Jiangjiang Bi^1^, Yilin Zhao^1^, Zhiqiang Zhou^1^, Shiyong Li^1^, Ailin Luo^1^

^1^Author affiliations: Department of Anesthesiology, Hubei Key Laboratory of Geriatric Anesthesia and Perioperative Brain Health, and Wuhan Clinical Research Center for Geriatric Anesthesia, Tongji Hospital, Tongji Medical College, Huazhong University of Science and Technology, 1095 Jiefang Avenue, Wuhan, 430030, Hubei, China.

^2^Author affiliations: State Key Laboratory of Oncology in Southern China, Department of Anesthesiology, Sun Yat-sen University Cancer Center, Guangzhou, 510060, Guangdong, China.

^3^Author affiliations: Department of Oncology, Tongji Hospital, Tongji Medical College, Huazhong University of Science and Technology, Wuhan 430030, China.

Corresponding authors: Shiyong Li, Ailin Luo

Email: shiyongli@hust.edu.cn (Shiyong Li), alluo@hust.edu.cn (Ailin Luo)

**
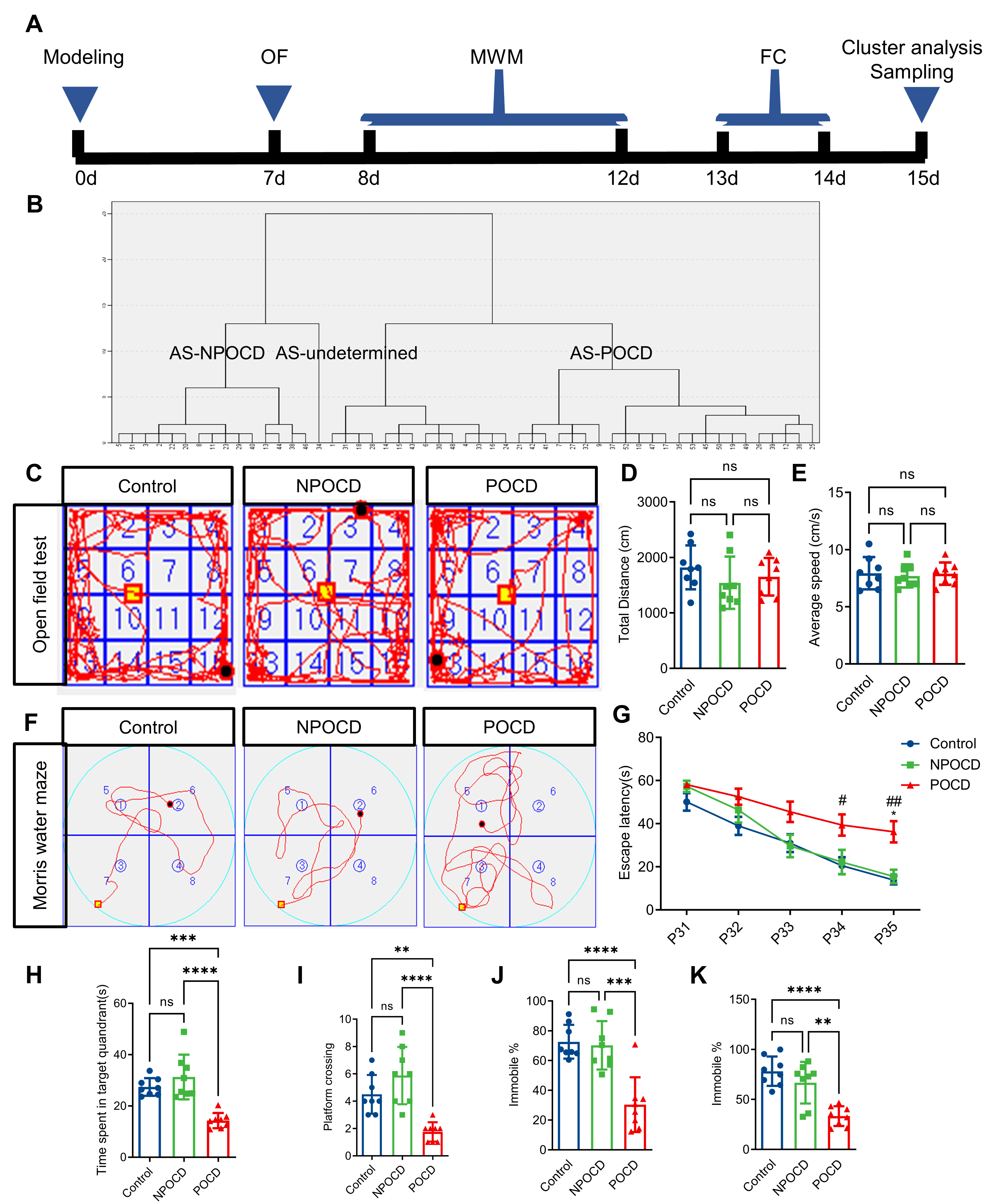
**

**Fig. S1 The elderly mice for whole transcriptome sequencing were divided into three groups (Control, NPOCD, and POCD) according to the behavioral tests.** (A) The timeline of the animal experiments was shown. The elderly male mice were performed with isoflurane anesthesia and intramedullary fixation for tibial fracture surgery. Then the tests of open field (OF), Morris water maze (MWM), and fear condition test (FCT) were subsequently conducted. (B) The data of MWM was clustered using the Euclidean distance measure with the Ward clustering algorithm. Mice who underwent anesthesia and surgery were split into two groups including the NPOCD and POCD groups. (C) The tracks of open field tests between three groups were shown. (D and E) The total distance and average speed of the three groups showed no significant difference. (F) The tracks of MWM for three groups were shown. (G) The escape latency was significantly longer in the POCD group compared to the NPOCD and control group on the fifth day. For part G in the figure, # means control vs POCD and P<0.05, ## P<0.01, and * means NPOCD vs POCD and P<0.05. (H and I) The time spent in the target quadrant and platform crossings was significant in the POCD group. (J and K) The percentage of freezing time was significantly lower in the POCD group. n = 8 per group. The P values were determined by one-way ANOVA (D, E, H-K) or two-way ANOVA (G) with multiple comparison tests; * P<0.05, ** P<0.01, **** P<0.0001, ns means P>0.05 (D, E, H-K).


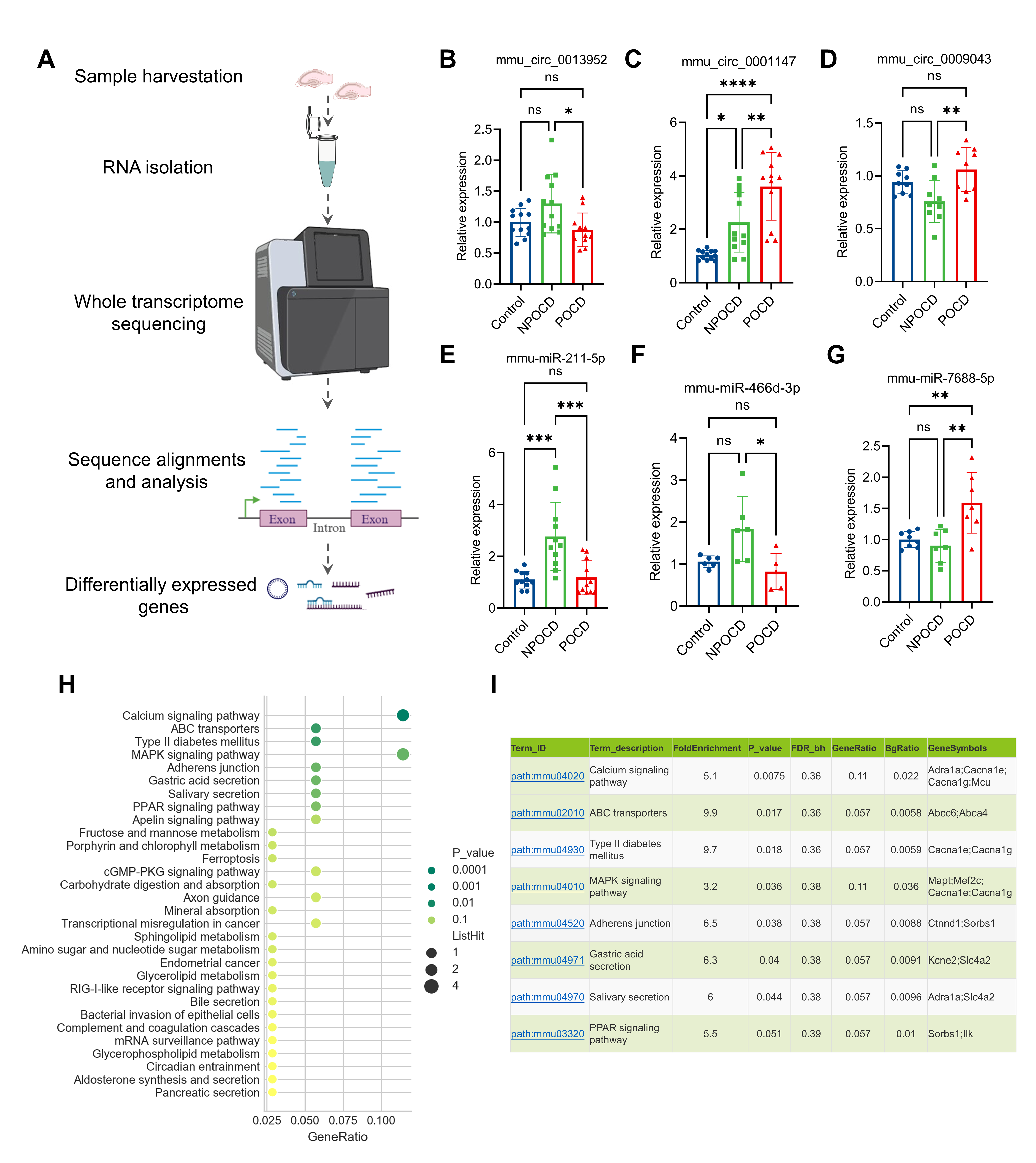


**Fig. S2 The hippocampi from mice of NPOCD and POCD groups were performed with whole transcriptome sequencing and analysis.** (A) The hippocampi from two mice were collected as one sample and three samples of each group were performed with whole transcriptome sequencing. (B-G) For the validation of profiles of differentially expressed genes, qPCR showed a significantly increased circ_0001147, circ_0009043 as well as miR-7688-5P, and decreased circ_0013952, miR-211-5p, miR-466d-3p in POCD group. In detail, the RNA for reverse transcription of circRNA was digested with RNase R. (H) Kyoto Encyclopedia of Genes and Genomes (KEGG) pathway analysis of circRNAs’ competitive endogenous RNA revealed the enriched pathway in MAPK signaling pathway using the hypergeometric distribution. (I) KEGG pathway analysis suggested that MEF2C would be involved in the MAPK signaling pathway. The P values were determined by one-way ANOVA with multiple comparison tests; * P<0.05, ** P<0.01, *** P<0.001, **** P<0.0001, ns means P>0.05.


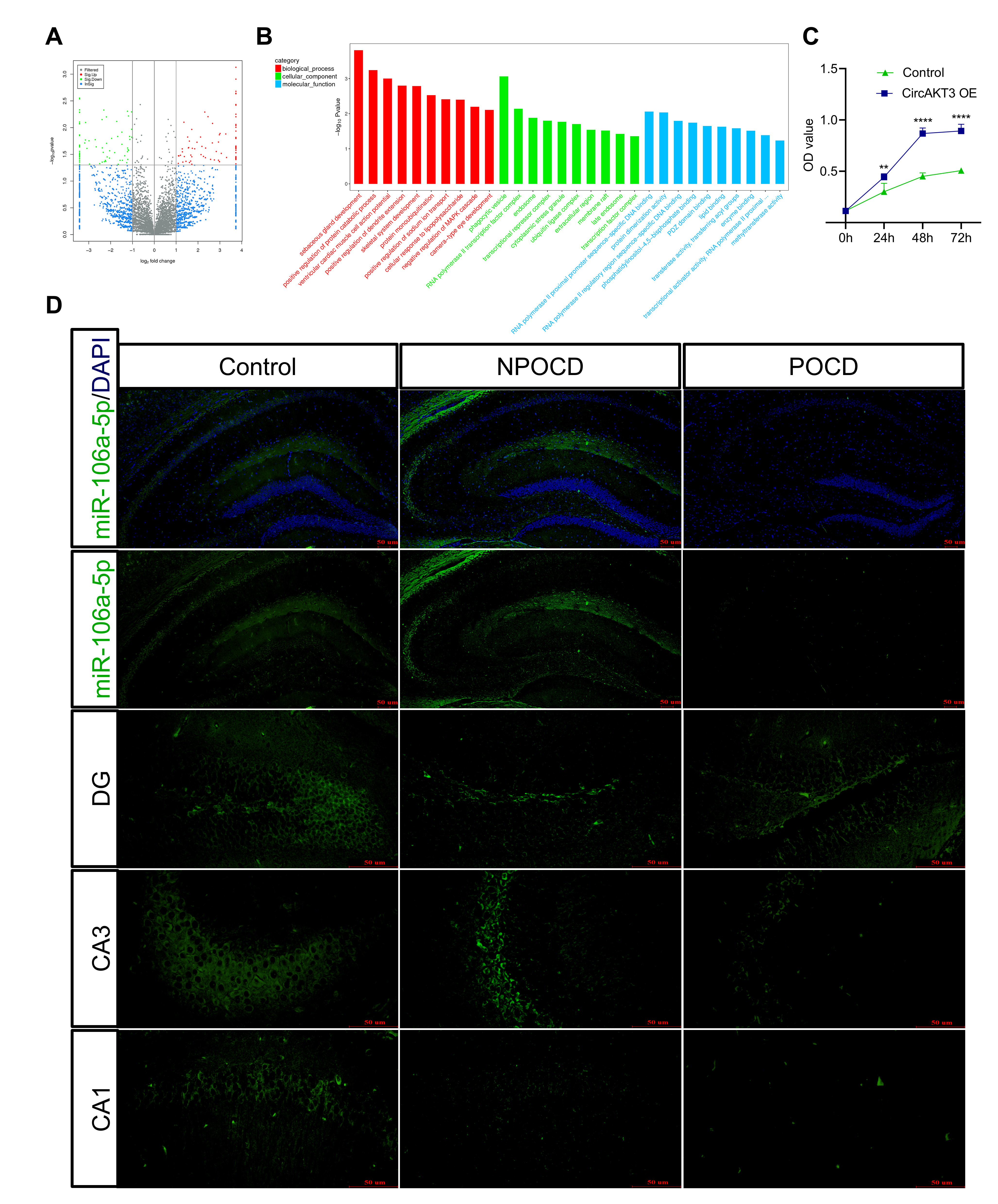


**Fig. S3 Gene Ontology (GO) functional enrichment analysis of circRNAs and miR-106a-5p was predominantly decreased in the cytoplasm of CA3 neurons.** (A) The volcano of differentially expressed circRNA was shown (filter: log_2_FC>1 or log_2_FC<-1). (B) GO functional enrichment analysis suggested that differentially expressed circRNA was enriched in the MAPK signaling pathway. (C) The cell viability was notably higher at 24h, 36h, and 48h in circAKT3-OE cells after Lipopolysaccharide (LPS) treatment (100ng/ml, 12 hours) compared to control cells. (D) FISH of miR-106a-5p discovered that miR-106a-5p was mainly localized in the cytoplasm of neurons in the hippocampal CA3 region. The scale bar was 50μm. The P values were determined by two-tailed unpaired Student’s t-test; ** P<0.01, *** P<0.001.


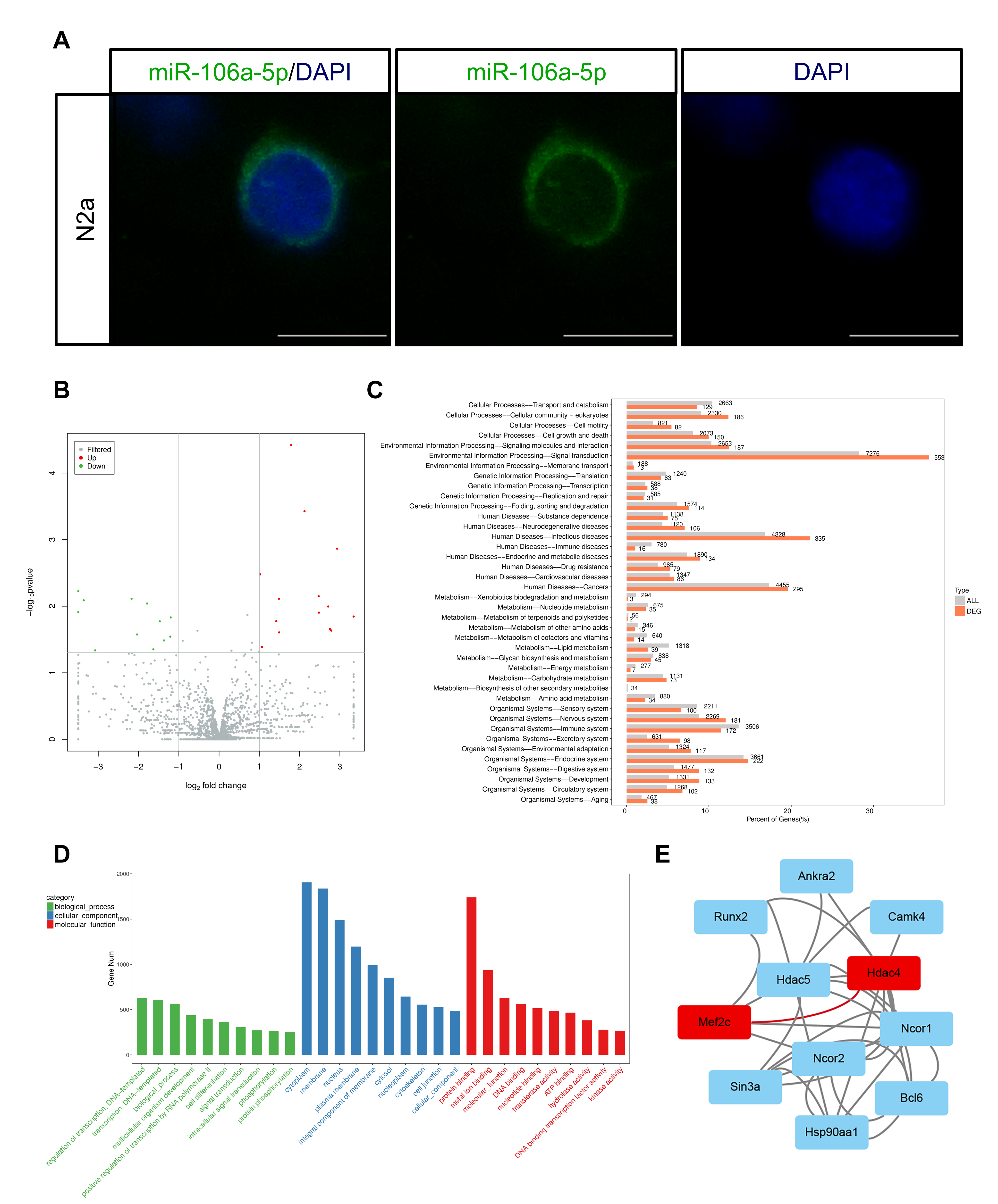


**Fig. S4 The localization of miR-106a-5p in N2a cells and KEGG, GO enrichment analysis of miRNA targets.** (A) FISH revealed that miR-106a-5p was mainly expressed in the cytoplasm of N2a cells. (B) The volcano of differentially expressed miRNA was shown (filter: log_2_FC>1 or log_2_FC<-1). (C) KEGG classification of differentially expressed miRNA targets revealed that environmental information processing was mostly enriched. (D) The biological process of GO enrichment analysis showed regulation of DNA-templated transcription. (E) String revealed the potential interactions of HDAC4 and MEF2C.


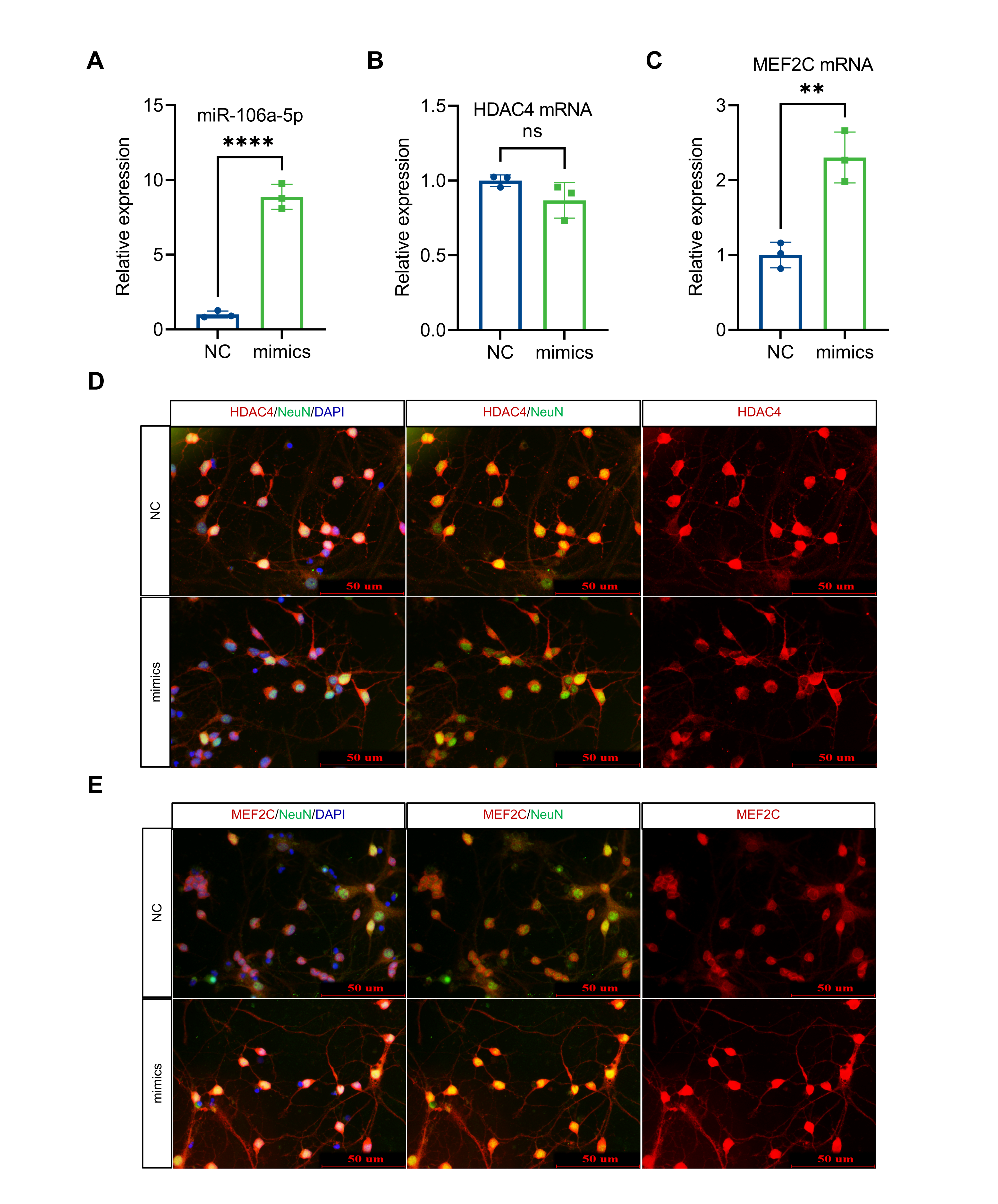


**Fig. S5 miR-106a-5p mimics reduced the level of HDAC4 protein and increased the level of MEF2C mRNA as well as protein in the primary neuron.** (A) qPCR validated the overexpression of miR-106a-5p upregulated by mimics. (B) qPCR showed that miR-106a-5p mimics did not affect the level of HDAC4 mRNA in the primary neuron. (C) qPCR showed that miR-106a-5p mimics significantly increased the level of MEF2C mRNA in the primary neuron. (D and E) Immunofluorescence revealed that mimics of miR-106a-5p notably reduced the fluorescence intensity of HDAC4 protein and increased the fluorescence intensity of MEF2C protein in primary neurons. The scale bar was 50μm. The P values were determined by two-tailed unpaired Student’s t-test; ** P<0.01, **** P<0.0001, ns means P>0.05.


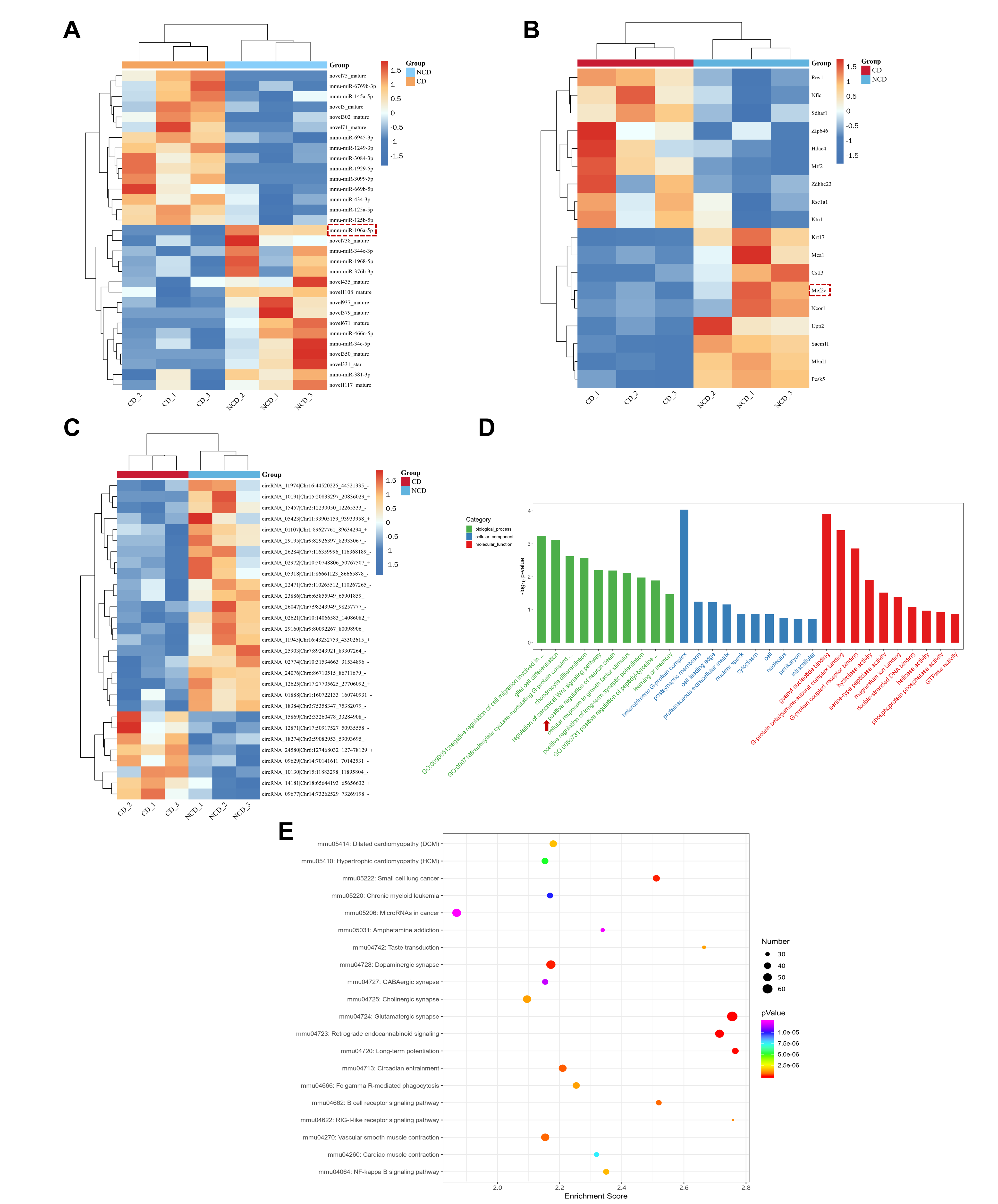


**Fig. S6 Differentially expressed gene profiles and analysis between preoperative cognitive disorders (CD) and non-cognitive disorders (NCD).** (A-C) The profiles of differentially expressed miRNA, mRNA, and circRNA between CD and NCD groups, were composed of 18-month-old male mice. (D) GO enrichment analysis of differentially expressed circRNAs indicated positive regulated neuronal death in the mice who underwent anesthesia and surgery (AS), compared to the mice without anesthesia and surgery. (E) KEGG analysis for differentially expressed miRNA targets revealed that long-term potentiation and glutamatergic synapses were most significantly enriched between AS and the control group.
